# Design Rule for Highly Stable Efficient High‐Entropy Metal Oxide Electrocatalysts: Complementary Roles of 3d Transition Metal Ions

**DOI:** 10.1002/advs.202510594

**Published:** 2025-09-16

**Authors:** Nam Hee Kwon, Woo Jin Noh, Seong‐Ju Hwang, Xiaoyan Jin

**Affiliations:** ^1^ Department of Materials Science and Engineering College of Engineering Yonsei University Seoul 03722 Republic of Korea; ^2^ Department of Battery Engineering Yonsei University Seoul 03722 Republic of Korea; ^3^ Department of Applied Chemistry University of Seoul Seoul 02504 Republic of Korea

**Keywords:** 3d transition metal substituent, electrocatalyst, electronic configuration, MnO_2_ nanowire, stability

## Abstract

The diversification of chemical composition has sparked significant research interest owing to its effectiveness in developing versatile functional high‐entropy materials. To develop highly stable, efficient metal oxide electrocatalysts, fundamental principles for the selection of efficient metal components must be established. In this study, the complementary roles of 3d transition metal components in enhancing the performance and stability of metal oxide electrocatalysts are systematically investigated. By examining the effects of single‐metal substitution on the electronic configuration and crystal morphology of MnO_2_ nanowires, V, Fe, Co, and Ni ions are identified as effective elements for improving electrocatalytic activity. The resulting quinary‐metal‐based α‐MnVFeCoNiO_2_ nanowires exhibited superior activity and stability for the oxygen evolution reaction (OER) over the unsubstituted α‐MnO_2_ and binary/ternary/quaternary‐metal‐based homologs. In situ Raman and density functional theory calculations demonstrated that multi‐metal substitution promoted the adhesion of the reaction intermediate during the OER. The improvements in OER performance can be attributed to the suppression of lattice oxygen occupation, the provision of diverse surface‐active sites, the enhancement of charge/mass transport, and the acceleration of electrocatalysis kinetics. Design factors are identified to be crucial for optimizing the electrocatalytic performance of high‐entropy MnO_2_ nanowires.

## Graphical abstract

Design rules for robust high‐performance metal oxide electrocatalysts are established by studying complementary roles of 3d transition metal ions. Based on the systematic investigation on the impact of metal substitution, several 3d metal ions are selected as optimized substituent ions. The resulting quinary α‐MnVFeCoNiO_2_ nanowires demonstrate outstanding electrocatalytic activity and durability due to the dynamic activation of surface active sites.

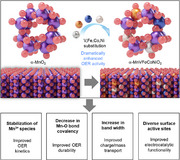

## 1 Introduction

High‐entropy materials featuring multi‐metallic compositions have garnered increasing research interest owing to their outstanding performance as energy‐functional and structural materials.^[^ ^1^, ^2^, ^3^ ^]^ The diversification of chemical composition effectively improves structural stability, regulates the electronic structure, and controls the nature of chemical bonding.^[^ ^4^, ^5^, ^6^ ^]^ As the chemical and physical characteristics of materials considerably influence their catalytic activity, the construction of high‐entropy multi‐metallic lattices can potentially facilitate the development of efficient and robust electrocatalysts.^[^ ^7^, ^8^ ^]^ Substitution with multi‐metal cations can increase bandwidth and can reduce the band gap, thereby improving charge‐transport properties.^[^ ^9^ ^]^ Additionally, diversifying metal compositions can allow for regulating the overlap between metal *n*d and ligand *n*p orbitals, thereby tuning the key factors influencing the operation mechanisms of electrocatalysis and enhancing the durability of high‐entropy electrocatalysts. Despite the advantage of high‐entropy introduction, most of the existing studies relied on trial‐and‐error methods,^[^ ^10^ ^]^ which limited the efficacy of this approach. Thus, to maximize the benefits of high‐entropy introduction in improving the functionalities of metal compounds, rational design principles for selecting appropriate metal substituents must be established. In this context, systematic investigations into the versatile effects of 3d transition metal elements on the electronic configuration, crystal morphology, and electrocatalytic functionality of metal oxides can provide valuable insights for guiding the design and synthesis of efficient high‐entropy materials.

Manganese oxides represent highly promising pristine materials for constructing high‐entropy lattices owing to their diverse crystal structures and high tolerance to multi‐metal substitution.^[^ ^11^, ^12^ ^]^ In addition, manganese oxides can form highly anisotropic 1D nanowires with versatile catalytic and electrode functionalities.^[^ ^13^, ^14^ ^]^ The elevated surface‐to‐volume ratio of highly anisotropic 1D nanowires allows substituted metal ions to be exposed at the surface, where they can serve as reaction centers, enriching catalytically active sites and accelerating electrocatalytic kinetics.^[^ ^15^, ^16^ ^]^ Thus, 1D manganese oxide nanowires may serve as effective substitution matrices for the synthesis of high‐entropy materials.^[^ ^17^, ^18^ ^]^ Among various manganese oxides, α‐MnO_2_ exhibits the highest electrocatalytic activity for the oxygen evolution reaction (OER).^[^ ^19^ ^]^ However, the insufficient durability of MnO_2_‐based electrocatalysts hinders the commercial use of these materials.^[^ ^19^ ^]^ Although the OER is crucial to the implementation of emerging energy technologies such as metal−air batteries and water electrolyzers,^[^ ^20^, ^21^, ^22^ ^]^ its sluggish kinetics prevent the commercialization of these technologies. Noble‐metal‐based catalysts, such as RuO_2_ and IrO_2_, exhibit excellent OER performance.^[^ ^23^ ^]^ However, they are characterized by high costs and limited availability, motivating the search for alternative electrocatalysts.^[^ ^24^ ^]^ In this context, multi‐metal substitution into α‐MnO_2_ nanowires offers a valuable opportunity for developing cost‐effective, high‐performance, highly stable electrocatalysts via the construction of high‐entropy lattices. Furthermore, a systematic investigation into the effects of different 3d transition metal substituents on the crystal structure, crystal morphology, and electronic structure of α‐MnO_2_ nanowires can provide useful insights to guide the design of efficient and robust transition‐metal‐based electrocatalysts. In situ spectroscopic analysis of composition‐optimized high‐entropy materials during electrocatalysis can help clarify the mechanisms responsible for the impact of high‐entropy introduction on the electrocatalytic performance and stability. However, to the best of our knowledge, there has been no prior study exploring the versatile roles of 3d transition metal elements in the synthesis of high‐entropy multi‐metal‐substituted MnO_2_ nanowires with efficient electrocatalytic functionality.

To address this gap, this study was aimed at establishing a versatile synthetic route for 1D high‐entropy metal oxide nanowires, based on co‐crystal growth from composition‐tunable ion‐adduct precursors containing a series of 3d transition metal ions (e.g., Sc, Ti, V, Cr, Mn, Fe, Co, Ni, Cu, and Zn). The influences of different 3d transition metal components on the crystal structure, crystal morphology, and electronic structure of α‐MnO_2_ were systematically examined using microscopic and spectroscopic investigations. The composition‐optimized quinary‐metal‐based α‐MnVFeCoNiO_2_ nanowires, as well as pristine α‐MnO_2_ and binary/ternary/quaternary‐metal‐based homologs, were evaluated as electrocatalysts toward the alkaline OER to highlight the advantages of cation diversification in improving the electrocatalytic activity and durability of metal oxides. The evolution of the chemical bonding nature of α‐MnVFeCoNiO_2_ nanowires during the OER was investigated using in situ Raman spectroscopy and density functional theory calculation to clarify the mechanism by which composition diversification enhances electrocatalyst activity.

## 2 Results and Discussion

### 2.1 Impact of 3d Transition Metal Substitution on Crystal Structure, Morphology, and Electrocatalytic Activity of Binary α‐Mn_1‐x_M_x_O_2_ 1D Nanowires

**Figure 1:**
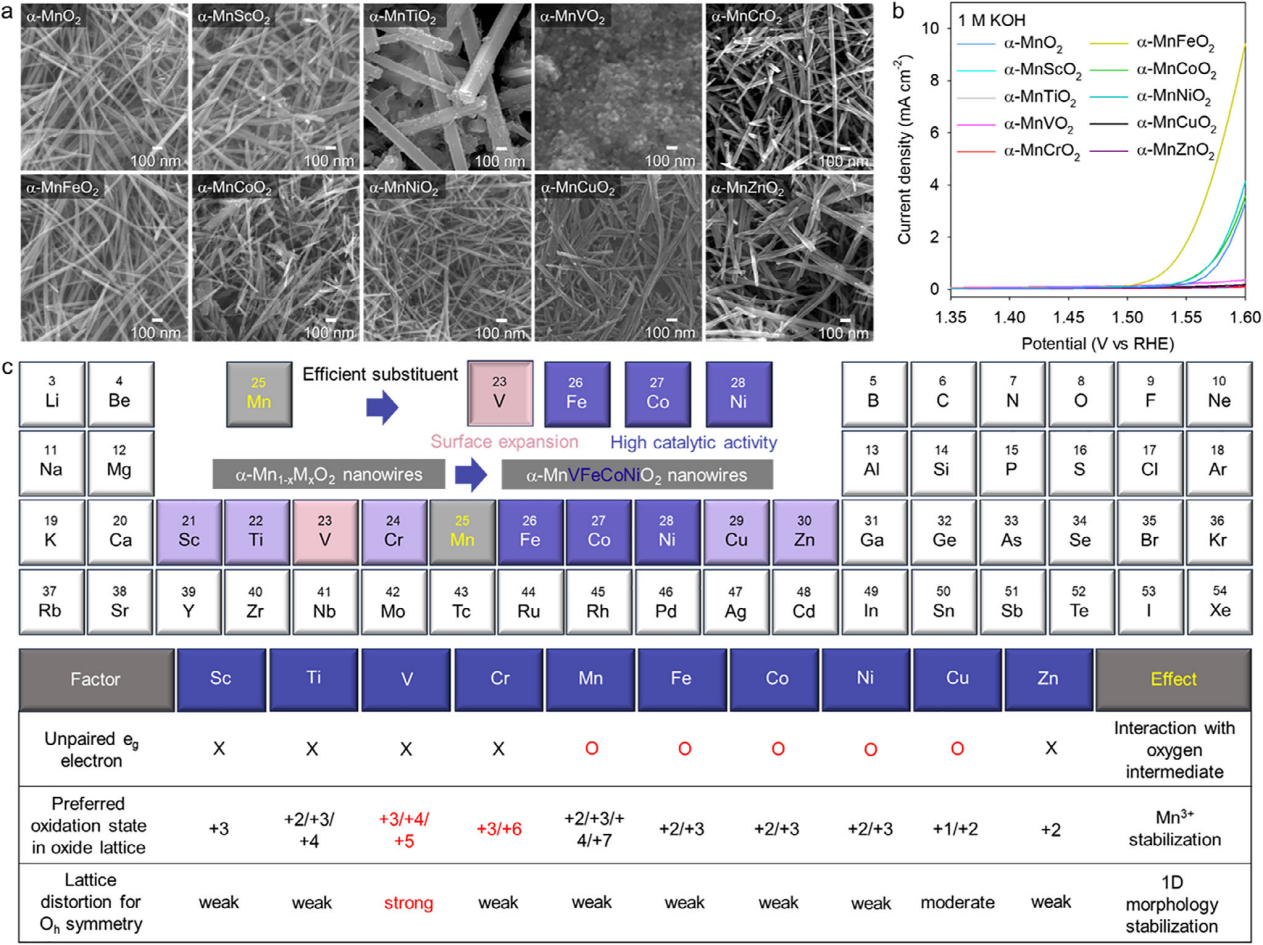
a) Powder XRD patterns, b) FE‐SEM images, and c) list of substituent elements for α‐MnO_2_ (with color coding) and the chemical properties of various 3d transition metals.

The effects of 3d transition metal substitution on the crystal structure of α‐MnO_2_ nanowires were examined through powder X‐ray diffraction (XRD). As shown in Figure S1 (Supporting Information), regardless of the type of 3d transition metal ion, all synthesized α‐Mn_1−x_M_x_O_2_ materials (M = Sc, Ti, V, Cr, Fe, Co, Ni, Cu, and Zn) exhibited the typical Bragg reflections of the tunnel‐structured α‐MnO_2_ phase without any impurity peaks, confirming the successful substitution of the metal ions in the α‐MnO_2_ lattice. The distinct variations in crystal morphology of α‐MnO_2_ upon the substitution of 3d transition metal ions were verified by field‐emission scanning electron microscopy (FE‐SEM) measurements (**Figure** **1**a). Although most of binary α‐Mn_1−x_M_x_O_2_ materials retained the original 1D nanowire morphology of α‐MnO_2_, the substitution of V ions decreased the nanowire length, resulting in a reduction in the aspect ratio. This marked frustration of α‐MnO_2_ nanowire growth upon V substitution could be ascribed to the strong preference of V ions for distorted local geometries with coordination numbers of 3, 4, and 5.^[^ ^25^ ^]^

The synthesized binary α‐Mn_1−x_M_x_O_2_ materials were used as electrocatalysts for the OER to clarify the influence of substituent type on electrocatalytic activity (Figure 1b). Among the binary α‐Mn_1−x_M_x_O_2_ materials, the Fe‐, Co‐, and Ni‐substituted nanowires showed promising electrocatalytic activity, highlighting their useful roles as substituents. As shown in Figure 1c, these results could be explained based on the electronic configurations of 3d metal ions. Early 3d transition metal ions, such as Sc, Ti, V, and Cr, do not have unpaired electrons in the *e*_g_ orbitals in their trivalent or tetravalent oxidation states. As unpaired electrons in *e*_g_ orbitals play a key role in the surface attachment of oxygen‐containing intermediates during the OER, these early 3d metal ions likely do not have appropriate electronic configurations to function as effective substituents. Conversely, late 3d transition metal ions such as Fe, Co, and Ni have unpaired *e*_g_ electrons in their divalent or trivalent oxidation states. Thus, these substituent ions can function as active sites for the OER. Although Cu^2+^ ions also have an unpaired electron in the *e*_g_ orbital, their strong preference for the low divalent oxidation state tends to reduce the Mn^3+^ concentration while increasing the Mn^4+^ concentration to maintain charge neutrality. Given that Mn^3+^ species are known to play a crucial role in the OER activity of MnO_2_ materials,^[^ ^26^ ^]^ the decrease in Mn^3+^ content following Cu^2+^ substitution can explain the inferior activity of α‐Mn_1−x_Cu_x_O_2_ (Figure 1c). Similarly, the strong preference of Zn to the divalent oxidation state and its completely occupied *e*_g_ configuration render Zn^2+^ ions inappropriate substituents for high‐entropy MnO_2_ materials (Figure 1c). In addition to these electronic factors, morphological factors also contributed to the OER activity of cation‐substituted MnO_2_ nanowires. Specifically, the shortening of 1D nanowires upon V substitution resulted in the expansion of surface area and the provision of additional surface‐active sites in α‐Mn_1−x_V_x_O_2_ materials, which might enhance the electrocatalytic functionality (Figure 1c). Based on these experimental findings, we selected V, Fe, Co, and Ni ions as substituents to synthesize high‐performance high‐entropy α‐MnO_2_ nanowires.

### 2.2 Impact of Multi‐metal Substitution on Crystal Structure of High‐entropy α‐MnVFeCoNiO_2_ 1D Nanowires

**Figure 2:**
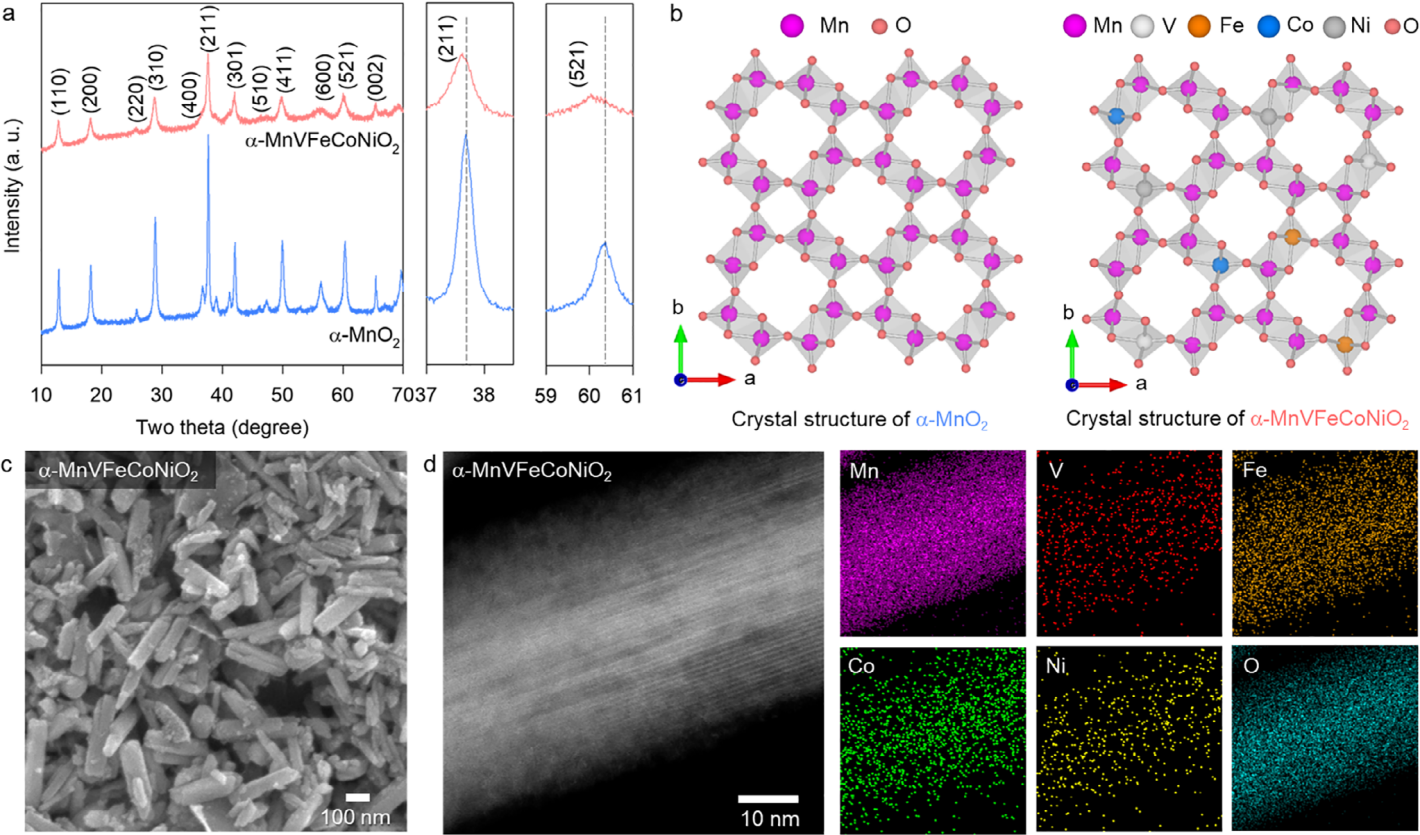
a) Powder XRD patterns, b) crystal structures, c) FE‐SEM image, d) TEM−elemental mapping data of α‐MnVFeCoNiO_2_ and α‐MnO_2_.

Based on the systematic investigation on the impact of substituent metal type on the properties of α‐Mn_1−x_M_x_O_2_, a quinary‐metal‐based α‐MnVFeCoNiO_2_ material was synthesized. As shown in **Figure** **2**a, the XRD pattern of α‐MnVFeCoNiO_2_ exhibited typical characteristics of the tunnel‐structured α‐MnO_2_ phase without any impurity peaks. This observation confirmed the successful substitution of all substituent metal ions in the manganese oxide lattice (Figure 2b). Compared with the unsubstituted α‐MnO_2_ material, this high‐entropy homolog showed a shift in the XRD peaks toward lower angles, reflecting an increase in the lattice parameters. The observed lattice expansion could be ascribed to the replacement of smaller Mn^3+^ (r = 0.645 Å) and Mn^4+^ (r = 0.53 Å) ions with larger Fe^3+^ (r = 0.645 Å), Co^2+^ (r = 0.65 Å), and Ni^2+^ (r = 0.69 Å) ions.^[^ ^27^ ^]^ Additionally, because the lattice parameters of α‐MnO_2_ are strongly dependent on the average oxidation state of Mn ions,^[^ ^17^ ^]^ the observed expansion of the unit cell suggests a reduction in the valence state of Mn ions upon multi‐metal substitution. This substitution‐induced lattice expansion was cross‐confirmed via least‐squares fitting analysis (Table S1, Supporting Information).

The intensity of the Bragg reflections decreased after multi‐metal substitution, indicating reduced crystal order in the α‐MnO_2_ lattice. This result could be attributed to the characteristic lattice distortion arising from the random distribution of multiple elements within the same crystal lattice, i.e., a fundamental feature of high‐entropy materials. The enhanced lattice distortion upon cation diversification was supported by the calculation of crystal strains based on Williams−Hall equation (Figure S2, Supporting Information).^[^ ^28^ ^]^ The α‐MnVFeCoNiO_2_ showed larger crystal strain than that of α‐MnO_2_ due to the random distribution of diverse substituent ions, which was responsible for the enhanced lattice distortion of this high‐entropy material.

FE‐SEM data demonstrated that both the unsubstituted α‐MnO_2_ and high‐entropy α‐MnVFeCoNiO_2_ materials exhibited a 1D nanowire morphology (Figures 1a and 2c). However, the aspect ratio of α‐MnVFeCoNiO_2_ was smaller than that of pristine α‐MnO_2_. This morphological change could be ascribed to the disruption of the crystal growth of MnO_2_ via the substitution of vanadium cations. This was confirmed by transmission electron microscopy (TEM) analysis, as shown in Figure S3a (Supporting Information). The high‐resolution TEM image revealed a slightly larger lattice fringe spacing in α‐MnVFeCoNiO_2_ compared to α‐MnO_2_, confirming the increased lattice parameter of the high‐entropy material, which is consistent with the XRD results (Figure S3b, Supporting Information). Moreover, this morphological change upon multi‐metal substitution was consistent with the weakening and broadening of the XRD peaks (Figure 2a). The homogenous substitution of multi‐metal cations (e.g., V, Fe, Co, and Ni) in manganese oxide was clearly substantiated by energy‐dispersive spectroscopy (EDS)−elemental mapping analysis, which revealed a uniform distribution of these metal elements throughout the nanowires (Figure 2d). Additionally, the ICP analysis was conducted to quantitatively determine the cation composition of α‐MnVFeCoNiO_2_, showing the Mn:V:Fe:Co:Ni ratio of 1.3 : 0.07 : 0.07 : 0.03 : 0.005. Notably, the concentration of Ni is considerably lower than the theoretical value, because the significant instability of high‐valent Ni^3+^/Ni^4+^ ions makes inefficient the incorporation of Ni into the Mn^4+^ octahedral sites. Also, the low stability of Co^4+^ oxidation state was responsible for the limited incorporation of Co ions in the high‐entropy α‐MnVFeCoNiO_2_ lattice. In contrast, V and Fe can be readily stabilized in the +4 state, resulting in the efficient substitution of these ions. The significant effects of multi‐metal substitution on the porosity of α‐MnO_2_ were confirmed through N_2_ adsorption−desorption isotherm analysis. Surface area calculations showed that α‐MnVFeCoNiO_2_ exhibited a significantly higher Brunauer−Emmett−Teller (BET) surface area of 89.1 m^2^ g^−1^ compared with that of α‐MnO_2_ (45.9 m^2^ g^−1^), highlighting the benefit of high‐entropy introduction in increasing the porosity (Figure S4, Supporting Information).

### 2.3 Impact of Multi‐Metal Substitution on the Chemical Bonding Nature of High‐Entropy α‐MnVFeCoNiO_2_ 1D Nanowires

**Figure 3:**
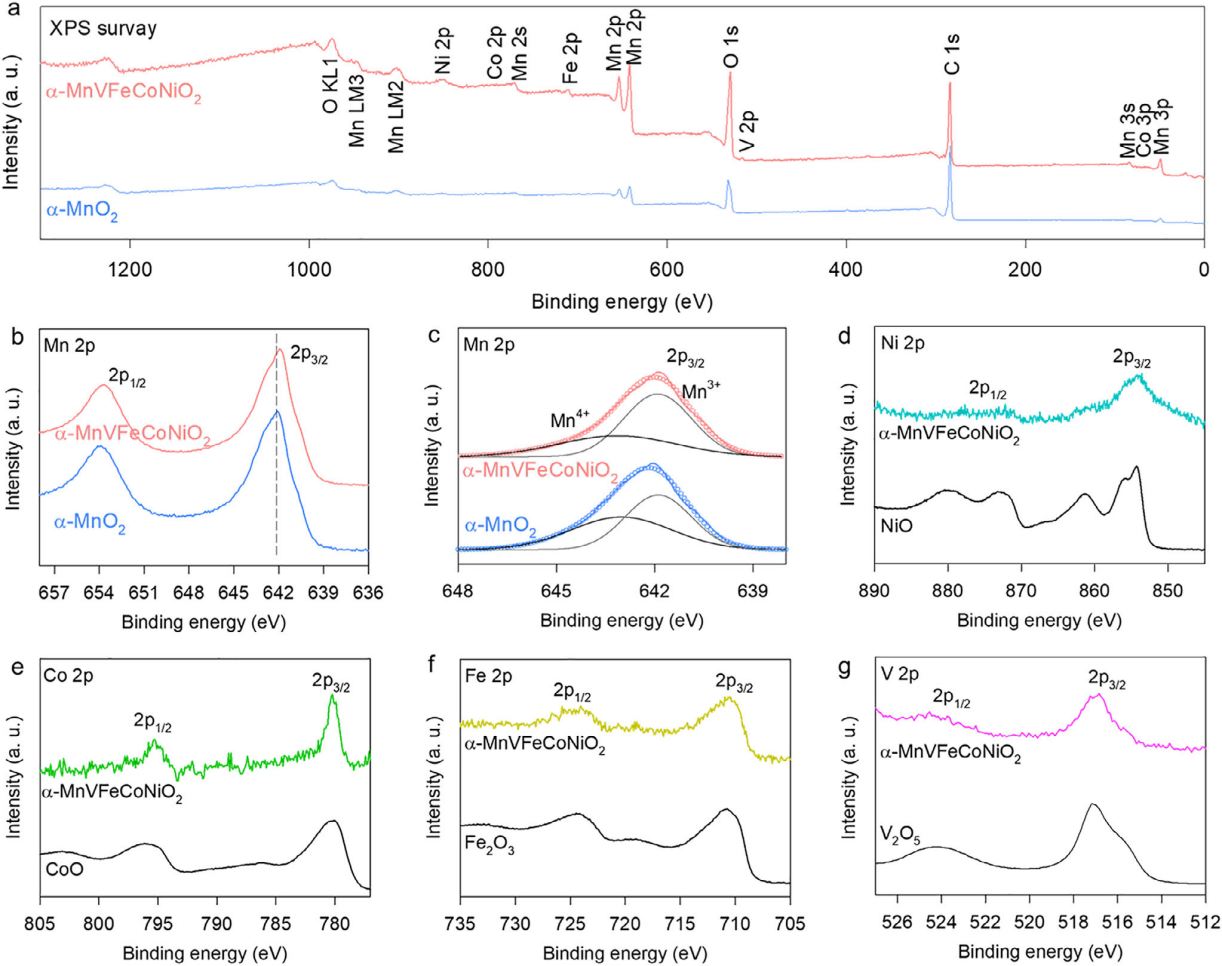
a) Survey XPS data, b) Mn 2p, c) peak deconvolution results of Mn 2p data, d) Ni 2p, e) Co 2p, f) Fe 2p, and g) V 2p XPS data of α‐MnVFeCoNiO_2_.

The influence of the diversified cation composition on the valence state of α‐MnVFeCoNiO_2_ was verified using X‐ray photoelectron spectroscopy (XPS). As shown in **Figure** **3**a, the survey XPS data confirmed the formation of the multi‐metal substituted α‐MnVFeCoNiO_2_. The Mn 2p XPS spectrum of α‐MnVFeCoNiO_2_ (Figure 3b) displayed typical features of α‐MnO_2_. According to the peak convolution analysis, the Mn 2p_3/2_ peak could be deconvoluted into two components corresponding to Mn^3+^ and Mn^4+^. As summarized in Figure 3c and Table S2 (Supporting Information), the content of Mn^3+^ increased by high‐entropy introduction, indicating a reduction in the average Mn valency. The comparison analysis with reference XPS data of V_2_O_5_, Fe_2_O_3_, CoO, and NiO in Figure 3 revealed that the V 2p, Fe 2p, Co 2p, and Ni 2p regions for α‐MnVFeCoNiO_2_ displayed typical spectral peaks of the V^5+^, Fe^3+^, Co^2+^, and Ni^2+^ valence states, respectively, confirming the synthesis of a high‐entropy material. Considering their electronic configurations, i.e., the presence of unpaired electrons in *e*_g_ orbitals (similar to the Mn^3+^ (d^4^) ion), the substituent Fe^3+^ (d^5^), Co^2+^ (d^7^), and Ni^2+^ (d^8^) ions in the octahedral sites were expected to act as active centers for interacting with the oxygen intermediate species. Hence, the exposure of these substituent ions at the surface of α‐MnVFeCoNiO_2_ nanowires likely contributed to improved electrocatalytic activity. In contrast, the V^5+^ (d^0^) ion lacked unpaired electrons in the *e*_g_ orbital and thus its contribution to the enhanced electrocatalytic performance could be ascribed to the morphological factor not to the electronic factor.

**Figure 4:**
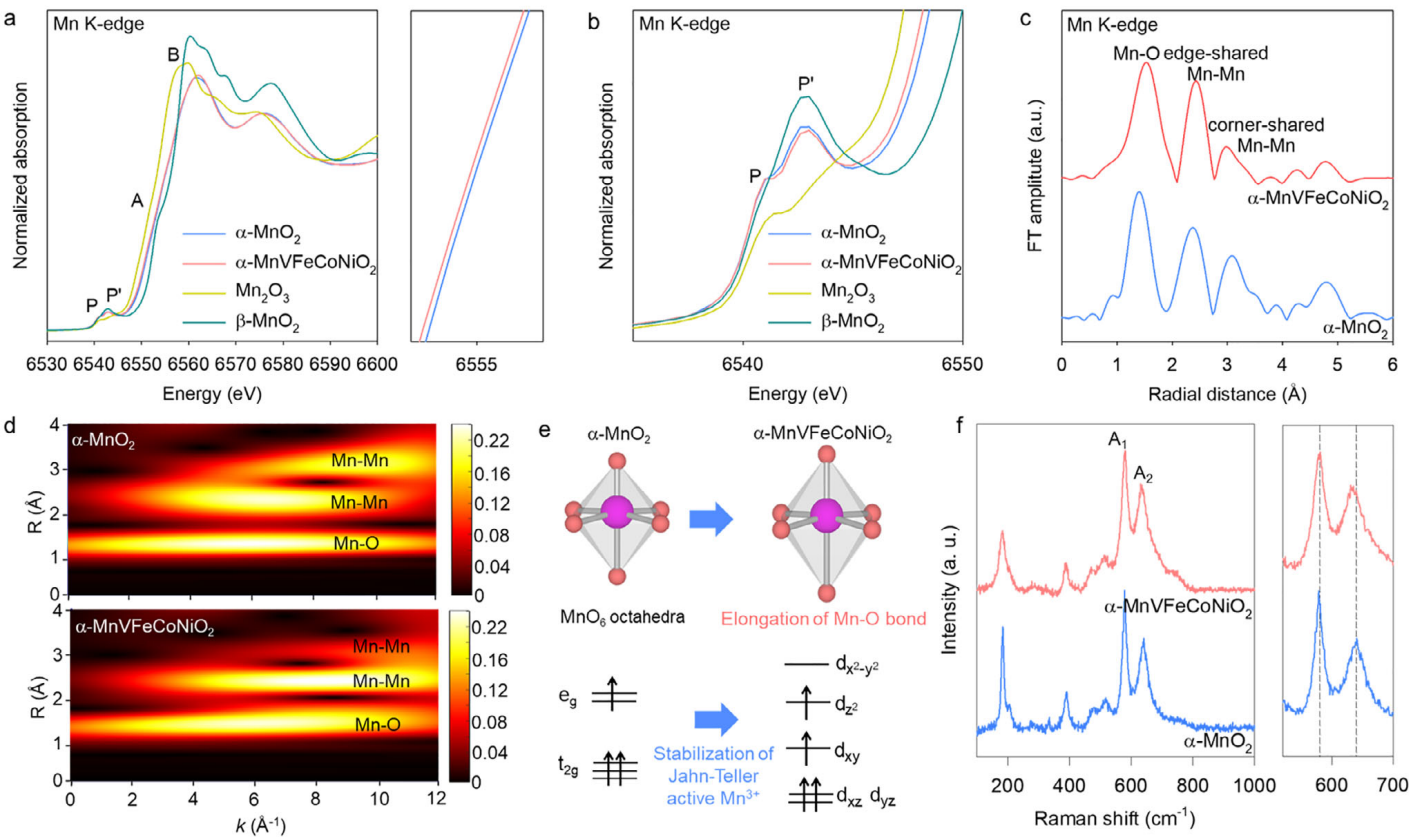
a) Mn K‐edge XANES spectra, b) enlarged view of pre‐edge spectra, c) Mn K‐edge FT‐EXAFS spectra, d) contour plot of the WT‐EXAFS data, e) local structural variation, and f) micro‐Raman data of α‐MnVFeCoNiO_2_, α‐MnO_2_, and references.

The influence of multi‐metal substitution on the local atomic geometry and bonding character around the Mn ions in α‐MnVFeCoNiO_2_ was corroborated by X‐ray absorption near‐edge structure (XANES) and extended X‐ray absorption fine structure (EXAFS) analyses. As depicted in **Figure** **4**a, both the unsubstituted α‐MnO_2_ and high‐entropy α‐MnVFeCoNiO_2_ exhibited the typical Mn K‐edge XANES spectral features of the α‐MnO_2_ phase, highlighting the retention of the α‐MnO_2_ lattice upon multi‐metal substitution. A closer inspection revealed that the edge energy of α‐MnVFeCoNiO_2_ was slightly lower than that of pristine α‐MnO_2_, stressing a reduction in the Mn oxidation state upon multi‐metal substitution. This observation was confirmed by the relative positions of the pre‐edge features P and P’ (Figure 4b), consistent with the enlarged unit cell volume for high‐entropy α‐MnVFeCoNiO_2_. To probe the local geometries and oxidation states of substituted transition metals, V K‐edge, Fe K‐edge, Co K‐edge, and Ni K‐edge XANES analyses were conducted (Figure S5, Supporting Information). These XANES spectra in the Fe K‐edge, Co K‐edge, and Ni K‐edge commonly displayed very weak pre‐edge peaks corresponding to the dipole‐forbidden 1s → 3d transitions, clearly indicating that Ni, Fe, and Co are stabilized in an octahedral coordination environment not in interstitial tetrahedral sites. This result is well‐consistent with the increase in lattice parameters, because the octahedral Fe^3+^, Co^2+^, and Ni^2+^ ions possess larger ionic radii compared to Mn^3+^/Mn^4+^.^[^ ^27^ ^]^ In contrast, the V K‐edge XANES spectrum of V‐substituted α‐MnO_2_ demonstrated an intense pre‐edge peak related to the dipole‐forbidden 1s → 3d transition, strongly suggesting that the substituted V^5+^ ions might exist in interstitial tetrahedral sites and/or strongly distorted octahedral sites of α‐MnO_2_ lattice. This finding accords with the strong preference of V ions for the highly distorted geometry from regular octahedra.

To acquire quantitative information about the multi‐metal‐substitution‐induced structural modifications, EXAFS analysis was performed. As plotted in Figure S6 (Supporting Information), the *k* space oscillations remained nearly unchanged before and after multi‐metal substitution, substantiating that the high‐entropy introduction does not disrupt the overall crystal structure. Fourier‐transformed (FT) EXAFS data at Mn K‐edge (Figure 4c) indicated that α‐MnVFeCoNiO_2_ displayed typical features of the α‐MnO_2_ phase, including three strong FT features at ~1.5, ~2.5, and ~3.0 Å, attributable to Mn−O, edge‐shared Mn−Mn, and corner‐shared Mn−Mn coordination shells, respectively.^[^ ^29^ ^]^ The FT peaks of α‐MnVFeCoNiO_2_ appeared less intense, broader, and shifted toward higher *R*‐values compared with those of unsubstituted α‐MnO_2_, confirming the degradation of structural order with Mn−O bond elongation upon the substitution of diversified cations. EXAFS fitting analysis (Figure S7 and Table S3, Supporting Information) revealed that the Mn−O bond distance in α‐MnVFeCoNiO_2_ was notably longer than that in α‐MnO_2_, indicating the reduction of the Mn oxidation state upon multi‐metal substitution. The substitution‐induced decrease in the FT intensity was further substantiated by wavelet‐transformed‐EXAFS data (Figure 4d). As shown in Figure 4e, the partial reduction of tetravalent Mn^4+^ ions led to the creation of Jahn−Teller active Mn^3+^ ions. The resulting elongation of the axial Mn−O bond contributed to the increase in the local structural deformation.

The notable change in the local structure of α‐MnO_2_ upon multi‐metal substitution was further corroborated by micro‐Raman spectroscopy. As shown in Figure 4f, even after multi‐metal substitution, all materials, including the high‐entropy α‐MnVFeCoNiO_2_ nanowires, displayed characteristic phonon lines of the α‐MnO_2_ phase with two intense peaks (A_1_ at ≈585 cm^−1^ and A_2_ at ≈645 cm^−1^), indicating the retention of the α‐MnO_2_ structure following high‐entropy introduction.^[^ ^17^ ^]^ Considering the mixed Mn^3+^/Mn^4+^ valency of the materials, the peaks A_1_ and A_2_ could be assigned to the totally symmetric A_1g_ vibration modes of the undistorted Mn^4+^O_6_ octahedra and Jahn−Teller distorted Mn^3+^O_6_ octahedra, respectively.^[^ ^30^, ^31^ ^]^ The substitution of multi‐metal ions decreased the A_1_/A_2_ intensity ratio, further supporting the reduction in the average Mn valence state with increasing Mn^3+^ concentration. In addition, the substitution of multi‐metal cations led to the displacement of the A_2_ peak toward lower wavelengths, indicating weakening of the Mn^3+^−O bond. This bond weakening was attributable to the competitive effect of neighboring substituent metal−oxygen bonds with higher bond covalency. Conversely, the A_1_ peak showed only a weak shift after the substitution. The dissimilar peak shifts of both Raman peaks could be ascribed to the stronger bonding strength of Mn^4+^−O compared with that of Mn^3+^−O. The combined spectroscopic results (Figure 4e) confirmed that the Mn−O bond was elongated upon the introduction of high entropy with diverse metal substituent ions.

### 2.4 Benefits of Multi‐Metal Substitution on the Electrocatalytic Performance of High‐Entropy α‐MnVFeCoNiO_2_ 1D Nanowires

**Figure 5:**
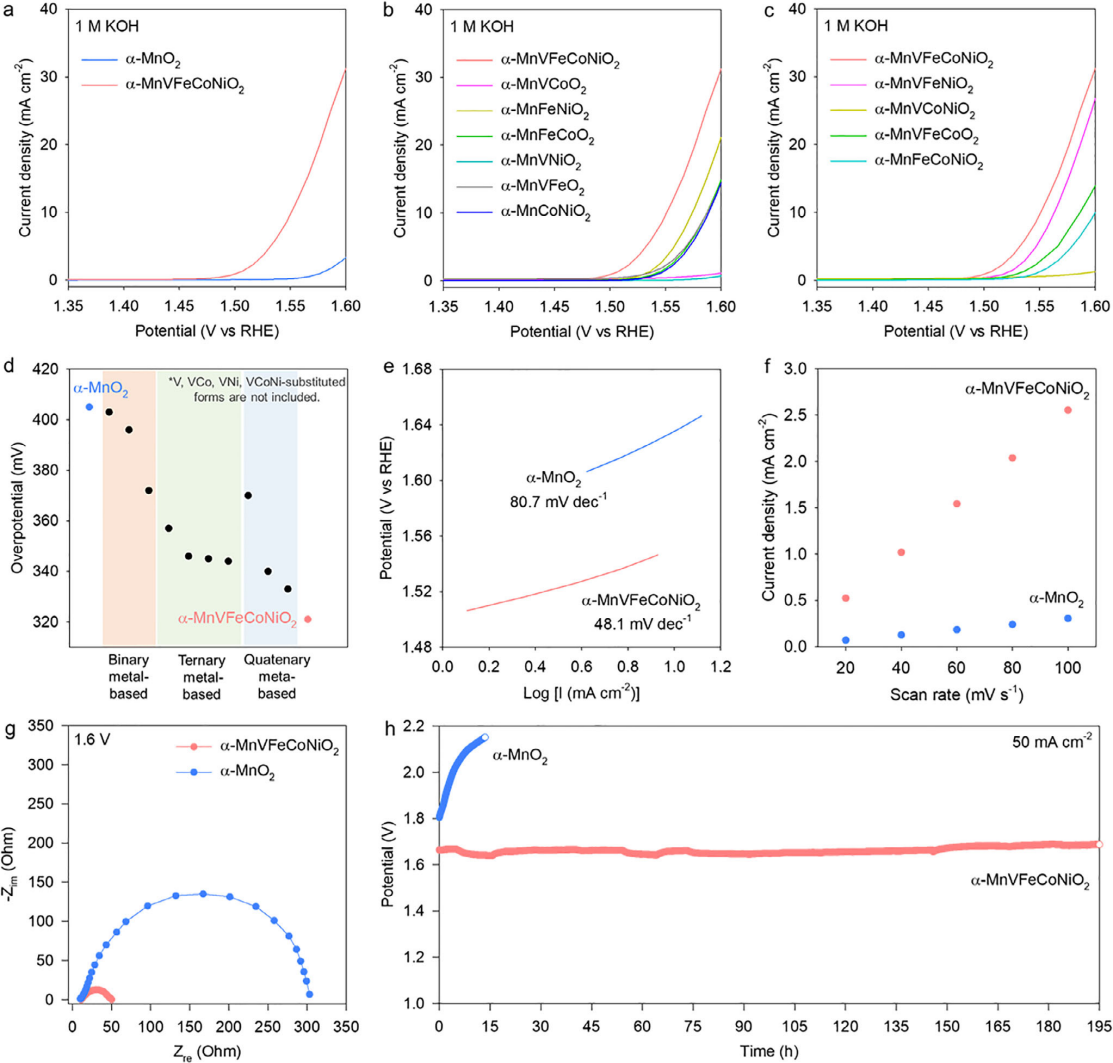
a−c) OER polarization curves, d) overpotential plots, e) Tafel slope, f) plots of current density difference versus scan rate, g) EIS data, and h) chronoamperometry curves for α‐MnVFeCoNiO_2_, α‐MnO_2_, and references.

To clarify the influence of high‐entropy introduction on the catalyst functionality of α‐MnO_2_, the electrocatalytic performance of α‐MnVFeCoNiO_2_ toward the OER in a 1 m KOH electrolyte was evaluated. Linear sweep voltammetry (LSV) curves (**Figure** **5**a) indicated that α‐MnVFeCoNiO_2_ delivered outstanding OER electrocatalytic activity, with a lower overpotential of 321 mV at 10 mA cm^−2^ and considerably larger current density compared with that of unsubstituted α‐MnO_2_ (overpotential of 405 mV). To further highlight the benefits of high‐entropy introduction on the electrocatalytic performance of α‐MnVFeCoNiO_2_, α‐MnO_2_ frameworks with binary, ternary, and quaternary metal compositions were synthesized and evaluated as OER electrocatalysts for comparison. XRD analysis (Figure S8, Supporting Information) confirmed the formation of α‐MnO_2_‐structured 1D nanowires of binary/ternary/quaternary‐metal oxides. Moreover, as shown in Figure 5a,c, the quinary metal‐based α‐MnVFeCoNiO_2_ displayed superior OER activity compared with the quaternary‐, ternary‐, and binary‐metal‐based α‐MnO_2_ nanowires.

Figure 5d illustrates the correlation between the overpotential and cation composition, indicating a gradual improvement in OER activity with increasing numbers of cation components. This observation provides convincing evidence for the effectiveness of high‐entropy introduction. Among the substituent ions, the incorporation of Fe^3+^, Co^2+^, and Ni^2+^, having unpaired electrons in the *e*_g_ orbitals, was quite effective in augmenting the advantage of cation diversification, as these ions efficiently acted as the catalytically active sites. In fact, the lower overpotential of high‐entropy α‐MnVFeCoNiO_2_ material (321 mV at 10 mA cm^−2^) is superior or comparable to the recently reported data of Fe‐, Co‐, or Ni‐doped MnO_2_ (Table S4, Supporting Information). Furthermore, while the overpotential of α‐MnVFeCoNiO_2_ material is comparable to that of commercial RuO_2_, the α‐MnVFeCoNiO_2_ material showed a much smaller Tafel slope than that of RuO_2_, indicating the excellent electrocatalytic activity of high‐entropy α‐MnVFeCoNiO_2_ toward OER. This comparison underscores the high efficacy of cation diversification in exploring the economically feasible electrocatalysts, see Figure S9 (Supporting Information). The benefit of high‐entropy introduction in promoting the OER catalysis kinetics was further supported by the considerably smaller Tafel slope of α‐MnVFeCoNiO_2_ (48.1 mV dec^−1^) compared with that of unsubstituted α‐MnO_2_ (80.7 mV dec^−1^), as shown in Figure 5e. Moreover, the turnover frequency (TOF) of the α‐MnVFeCoNiO_2_ was evaluated to be 0.77 s^−1^ at an overpotential of 400 mV, which is superior to that of α‐MnO_2_ (0.14 s^−1^), stressing the benefit of high‐entropy introduction in improving the electrocatalyst performance. From the CV data measured at various scan rates and plots of current density difference versus scan rate (Figure 5f; Figure S10, Supporting Information), the electrochemical surface area (ECSA) of α‐MnVFeCoNiO_2_ was determined to be 317.5 cm^2^, which is considerably larger than that of pristine α‐MnO_2_ (36.25 cm^2^). This substantial increase in ECSA upon the high‐entropy introduction contributes to the improved OER activity of α‐MnVFeCoNiO_2_. Electrochemical impedance spectra (EIS) data (Figure 5g) demonstrated that multi‐metal substitution markedly decreased the diameter of the semicircle measured at 1.6 V (versus reversible hydrogen electrode), indicating improved charge‐transport properties.^[^ ^32^ ^]^ According to the fitting analysis (Figure S11, Supporting Information), the charge‐transfer resistance (*R*_ct_) of α‐MnVFeCoNiO_2_ (38.1 Ω) was considerably lower than that of the pristine α‐MnO_2_ homolog (292.7 Ω), confirming the enhancement in electrical transport following high‐entropy introduction. This observation highlighted the effectiveness of cation‐composition diversification in improving charge‐transport properties, thereby enhancing the electrocatalytic performance of α‐MnVFeCoNiO_2_. In addition, in situ Bode plots were measured to further probe the variation of OER interfacial kinetics (Figure S12, Supporting Information). The in situ Bode plots clearly demonstrated the multi‐metal‐substitution‐induced change in OER interfacial kinetics.^[^ ^33^ ^]^ In the case of high‐entropy α‐MnVFeCoNiO_2_, the phase maximum peak showed the depression of intensity and the notable shift toward higher frequency as the potential increases, indicating the acceleration of charge‐transfer dynamics and the shortening of time constants. In contrast, unsubstituted α‐MnO_2_ retained a higher intensity of phase maximum with less frequency shift, reflecting its slower surface processes. These results demonstrate that α‐MnVFeCoNiO_2_ showed more favorable OER kinetics than α‐MnO_2_. Chronoamperometry results (Figure 5h) indicated that α‐MnVFeCoNiO_2_ exhibited outstanding durability over 195 h at 50 mA cm^−2^, whereas pristine α‐MnO_2_ suffered from significant degradation in the early stage of the stability test. Such excellent catalyst stability and activity of α‐MnVFeCoNiO_2_ were found to be superior over those of recently reported MnO_2_‐based materials.^[^ ^34^, ^35^, ^36^, ^37^, ^38^ ^]^ The improved stability of α‐MnVFeCoNiO_2_ was further corroborated by combined ex situ characterization after the long‐term OER test.^[^ ^39^, ^40^, ^41^ ^]^ As presented in Figure S13, the original features in the XRD and FE‐SEM data of α‐MnVFeCoNiO_2_ remained unchanged before and after the OER durability test, confirming the excellent electrostability of high‐entropy α‐MnVFeCoNiO_2_ nanowire. The present results provide convincing evidence for the high efficacy of high‐entropy introduction with finely selected substituent cations in improving the durability of MnO_2_‐based electrocatalysts.

In addition, the effect of the Mn/V/Fe/Co/Ni ratio on OER activity was examined by evaluating the electrocatalytic performance of α‐MnVFeCoNiO_2_ materials with various metal ratios to verify the validity of the selected substitution ratios. The dependence of OER activity on the metal ratio (Figure S14, Supporting Information) clearly demonstrated that the composition of Mn:(V+Fe+Co+Ni) = 0.8:0.2 was optimal for enhancing the electrocatalytic functionality.

### 2.5 In situ Raman Spectroscopic Analysis of High‐entropy α‐MnVFeCoNiO_2_ 1D Nanowires

**Figure 6:**
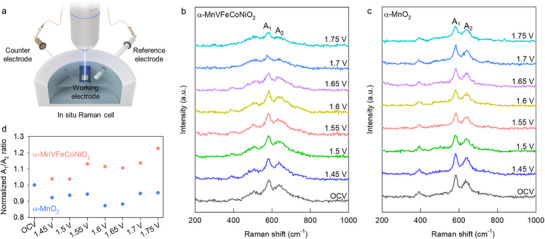
a) Schematic illustration for in situ Raman setup. In situ Raman data of b) high‐entropy α‐MnVFeCoNiO_2_ and c) pristine α‐MnO_2_ during the OER process. d) Variation of A_1_/A_2_ ratios as a function of applied potential.

To clarify the mechanism underlying the enhancement of OER activity by multi‐metal substitution in α‐MnO_2_ nanowires, the changes in the lattice vibrations of α‐MnVFeCoNiO_2_ during the OER were examined through in situ Raman spectroscopy. A custom‐designed in situ cell was fabricated to minimize background noise in Raman scattering, as displayed in **Figure** **6**a. Before applying an electrical potential, characteristic phonon lines of the α‐MnO_2_ phase, including two intense Raman peaks at ≈585 and ≈645 cm^−1^, were observed for both α‐MnO_2_ and α‐MnVFeCoNiO_2_,^[^ ^42^ ^]^ as shown in Figure 6b. The application of increasing oxidation potential (1.45−1.75 V) to α‐MnVFeCoNiO_2_ resulted in a significant decrease in intensity and broadening of the A_2_ peak (related to tetragonally distorted Mn^3+^O_6_ octahedra), demonstrating the variation in Mn^3+−^O bond strength. Given that Mn^3+^ considerably affects the OER activity of manganese oxide,^[^ ^43^ ^]^ the observed potential‐induced broadening of A_2_ provides strong evidence for the enhanced binding of the oxygen reaction intermediate to Mn^3+^ sites.

Conversely, as shown in Figure 6c, no significant change in A_2_ occurred for the unsubstituted α‐MnO_2_ upon increasing the oxidation electrical potential, revealing insignificant changes in the lattice vibration of α‐MnO_2_ and a lack of activation of Mn^3+^ sites during the OER. The significant influence of multi‐metal substitution on the surface reactivity was further substantiated by the greater change in the A_1_/A_2_ ratio for quinary‐metal‐based α‐MnVFeCoNiO_2_ compared with that for unsubstituted α‐MnO_2_. This observation provided additional proof for the effectiveness of high‐entropy introduction in activating the Mn^3+^ sites in the OER (Figure 6d), enhancing the electrocatalytic activity of manganese oxides.

### 2.6 DFT Calculation of High‐Entropy α‐MnVFeCoNiO_2_ 1D Nanowires

**Figure 7:**
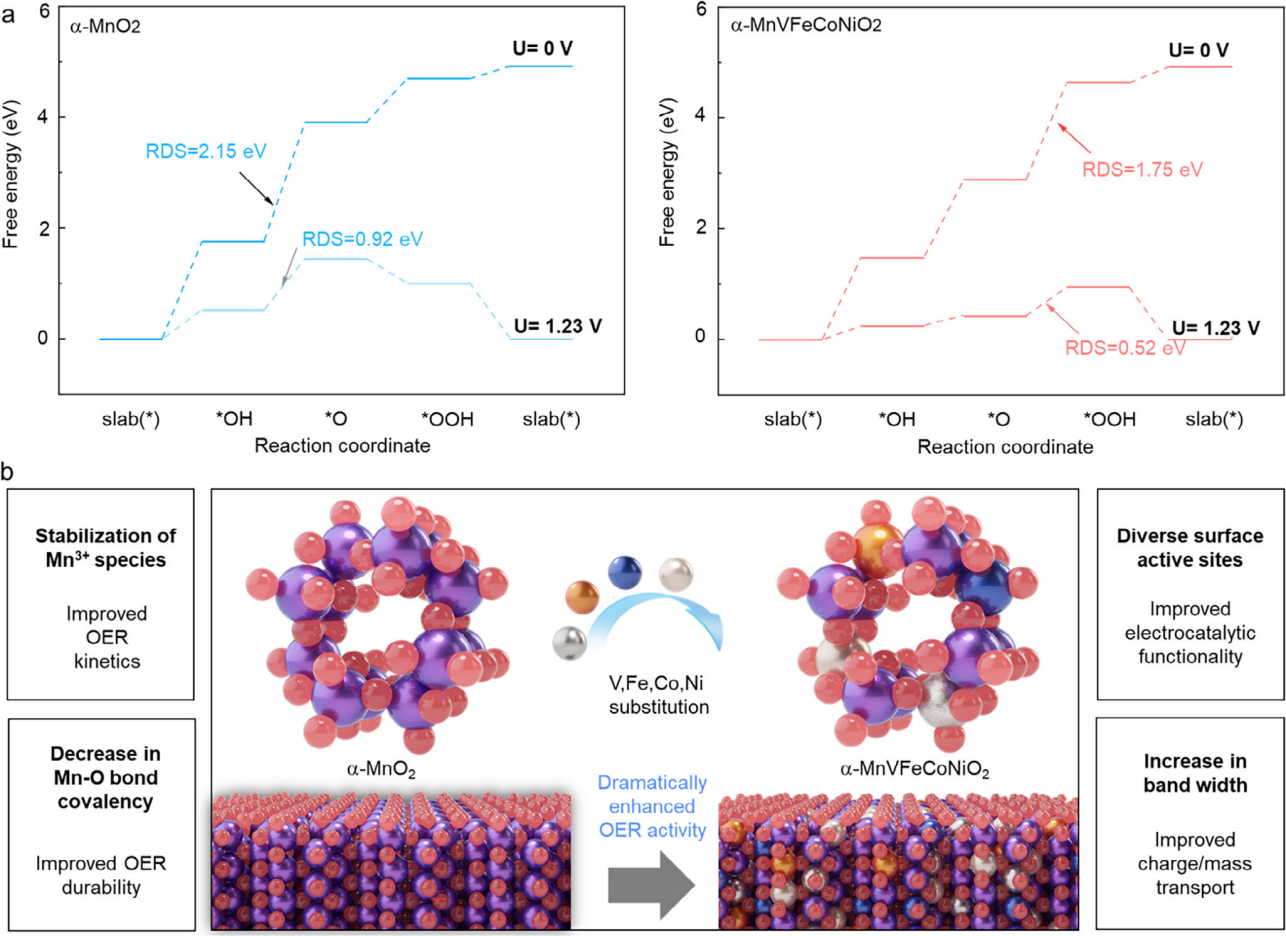
a) Gibbs free energy diagrams in OER process. b) Schematic illustration of several contributing factors of high‐entropy introduction to the improved electrocatalyst activity and durability of α‐MnVFeCoNiO_2_ nanowires toward the OER process.

To clarify the underlying mechanism for the enhancement of OER activity by multi‐metal substitution, density functional theory (DFT) calculations were performed to investigate the operation mechanisms of α‐MnO_2_ and α‐MnVFeCoNiO_2_ materials. As shown in **Figure** **7**a, the calculated free energy profiles revealed that multi‐metal substitution decreases the rate‐determining step (RDS) energy barrier from 2.15 to 1.75 eV at zero applied potential, indicating a significant reduction in activation energy. At an applied potential of 1.23 V, the high‐entropy α‐MnVFeCoNiO_2_ catalyst exhibits an even lower RDS free energy barrier of 0.52 eV compared to 0.92 eV for the α‐MnO_2_, highlighting its enhanced catalytic activity. This improvement could be attributed to the facilitated conversion from ^*^OH to ^*^O intermediates upon high‐entropy introduction, which leads to a more favorable reaction pathway. These results demonstrate that multi‐metal substitution effectively enhances the OER performance of α‐MnO_2_ by modifying the surface electronic properties and stabilizing the reaction intermediates.

In summary, several key factors contributed to the beneficial effects of high‐entropy introduction on the electrocatalytic OER activity of multi‐metal‐substituted α‐MnVFeCoNiO_2_ (Figure 7b). First, the stabilization of catalytically active Mn^3+^ sites in the high‐entropy α‐MnVFeCoNiO_2_ nanowire improved the OER kinetics through enhanced interactions with oxygen reaction intermediates, as supported by in situ Raman spectroscopic analyses. Second, the expansion of the unit cell volume upon high‐entropy introduction reduced the metal−oxygen bond covalency. The resulting decrease in the overlap of the metal 3d and oxygen 2p bands effectively diminished the contribution of the lattice oxygen occupation mechanism to the electrocatalytic activity of α‐MnVFeCoNiO_2_ nanowires, enhancing their OER durability. Third, the diversification of metal components resulted in the creation of diverse surface reaction sites. Substituted Fe^3+^ (d^5^), Co^2+^ (d^7^), and Ni^2+^ (d^8^) ions, with unpaired electrons in the *e*_g_ orbitals, functioned as active centers for interactions with the oxygen intermediate species. In contrast, the V^5+^ (d^0^) ions, lacking *e*_g_ electrons, were noted to be less effective as active centers. However, V^5+^ substitution led to a shortening of the nanowire morphology and an increase in the surface area (Figure 1a), which contributed to improved electrocatalytic functionality by promoting OH^−^ ion access to the surface reaction sites.^[^ ^44^ ^]^ Fourth, the stabilization of multi‐metal cations in the α‐MnVFeCoNiO_2_ increased the band width and decreased the bandgap energy, leading to improved charge/mass transport kinetics, which enhanced the OER activity of the α‐MnVFeCoNiO_2_ nanowires. In this regard, the exact role of different 3d metal substituents in this quinary α‐MnVFeCoNiO_2_ system can be defined as follows; 1) The V^5+^ ions could play the role of morphology modifiers by frustrating the crystal growth of 1D nanowires due to their strong preference to the highly distorted local geometry, leading to the increase in surface area. 2) Judging from the reported high activity of Fe^3+^ ion for OER,^[^ ^45^ ^]^ the substituted Fe^3+^ ions were supposed to act as catalytically active sites for the oxidation of H_2_O reactant. 3) Taking into account the larger ion sizes of divalent Co^2+^ and Ni^2+^ with respect to Mn^4+^, these substituent metal ions caused the increase in unit cell volume, indicating their role of lattice dilatants. The resulting lattice expansion led to the weakening of Mn─O bond covalency and hence to minimizing the redox of lattice oxygen, which was responsible for the improved durability of high‐entropy of α‐MnVFeCoNiO_2_. To assess the capital cost and the technique feasibility for preparing this complex quinary high‐entropy material, the production cost was calculated. The estimated cost of producing α‐MnVFeCoNiO_2_ system is ≈13 USD kg^−1^ (Table S5, Supporting Information). However, incorporating even a small amount of Ru or Ir significantly increases the material cost to ≈5141 and 120888 USD kg^−1^, respectively. This substantial cost difference clearly underscores the economic benefit of our high‐entropy α‐MnVFeCoNiO_2_ system, which entirely avoids the use of costly noble metals. In addition, the synthesis route we employed is based on a simple, scalable, and solution‐based process, demonstrating not only technical feasibility but also strong potential for industrial‐scale implementation. Taken together, this approach provides a promising balance of high electrocatalytic performance, cost‐efficiency, and practical manufacturability.

## 3 Conclusion

We proposed a high‐entropy introduction approach to develop robust and efficient electrocatalysts for the alkaline OER, relying on multi‐metal substitution in 1D α‐MnO_2_ nanowires. The systematic investigation about the impact of substituent type on the OER activity of binary α‐Mn_1−x_M_x_O_2_ nanowires allowed to select the V, Fe, Co, and Ni ions as efficient species for the development of efficient high‐entropy electrocatalysts. In fact, the diversification of metal composition with V, Fe, Co, and Ni substituent ions substantially enhanced the electrocatalytic performance of high‐entropy quinary‐metal‐based α‐MnVFeCoNiO_2_ nanowire toward the OER in a 1 m KOH alkaline electrolyte, surpassing those of pristine α‐MnO_2_ and binary/ternary/quaternary‐metal‐based homologs. Multi‐metal substitution in α‐MnO_2_ enhanced the structural stability of the catalytically active Mn^3+^ components and expanded the lattice parameters. The resulting elongation of the Mn─O bond upon high‐entropy introduction decreased the metal−oxygen bond covalency, which mitigated lattice oxygen occupation during the OER. This effect contributed to the outstanding electrocatalyst durability of the high‐entropy α‐MnVFeCoNiO_2_ nanowires. In situ Raman analysis conducted during the electrocatalytic reaction highlighted that the numerous Mn^3+^ sites strengthened the adhesion of the reaction intermediate, thereby enhancing the electrocatalytic performance. Additionally, the diversification of metal components in the α‐MnVFeCoNiO_2_ lattice decreased the bandgap energy and provided abundant surface active sites, leading to enhanced charge/mass transport, increased ECSA, and a ccelerated electrocatalysis kinetics. These factors contributed to the significant improvement in the electrocatalytic functionality of α0MnVFeCoNiO_2_ nanowires. Design factors such as occupancy of *e*_g_ orbitals, local geometry preferences, and stabilization of Mn^3+^ ions were identified to be crucial for optimizing the electrocatalytic performance of high‐entropy MnO_2_ nanowires. Given the effectiveness of cation diversification in enhancing the electrocatalytic activity and stability of metal oxides, the proposed high‐entropy introduction approach provides a promising pathway for developing high‐performance robust electrocatalyst materials for use in water electrolyzers and metal−air batteries.

## 4 Experimental Section

### Synthesis

High‐entropy 1D α‐MnVFeCoNiO_2_ nanowire was synthesized by the hydrothermal reaction of a homogeneous precursor solution of KMnO_4_, VCl_3_, Fe(NO_3_)_3_·9H_2_O, Co(NO_3_)_2_·6H_2_O, Ni(NO_3_)_2_·6H_2_O, and Na_2_S_2_O_8_ at 140 °C for 12 h. The molar ratio of Mn:V:Fe:Co:Ni was controlled to be 0.8:0.05:0.05:0.05:0.05. After completion of the synthesis, the obtained powders were thoroughly washed with distilled water and oven‐dried at 50 °C. For comparison, several binary/ternary/quaternary metal‐based α‐MnO_2_ nanowires were also synthesized under identical synthetic conditions to those used for the α‐MnVFeCoNiO_2_ nanowire.

### Sample Characterization

The influence of multi‐metal substitution on the crystal structures of α‐MnVFeCoNiO_2_ was characterized with XRD using a Rigaku Miniflex diffractometer (Cu K_α_ radiation). The morphological changes were studied by monitoring FE‐SEM (Jeol JSM‐7001F) images. TEM images of present materials were collected using Jeol JEM‐ARM200F microscope. The compositions and spatial distributions of cationic components were investigated by measuring EDS−elemental mapping data with focoused ion beam (FIB) cross‐sectioning. The impact of multi‐metal substitution on the bonding character was characterized by XPS using an XPS spectrometer (Thermo VG, UK, Al K_α_). Elemental analysis was performed using ICP‐OES (Agilent 5110). The electronic structures of the materials were probed with XANES spectroscopy, whereas EXAFS analyses were conducted to determine the local atomic arrangements of the materials. XANES/EXAFS spectra were recorded at the Pohang Accelerator Laboratory (10C beamline, Pohang, Republic of Korea). The measured energies of the collected spectra were referenced to the simultaneously measured reference data for the metal foils. The curve‐fitting analysis of the EXAFS data was performed using a well‐established standard procedure.^[^ ^46^ ^]^ In situ Raman analysis was conducted using a micro‐Raman spectrometer (Horiba Jobin Yvon LabRam Aramis), in which an Ar ion laser beam (λ = 514.5 nm) was utilized as an excitation source. N_2_ adsorption–desorption isotherm data were measured at 77 K using a MicrotracBEL BELSORP‐miniX analyzer.

### Electrocatalyst Functionality Test

The electrocatalytic OER functionality of the synthesized high‐entropy nanowires was tested by measuring LSV curves using an IVIUM analyzer with a three‐electrode cell. High‐entropy 3d transition metal substituted α‐Mn_1−x_M_x_O_2_ nanowires were employed as working electrodes, with a Pt wire as the counter electrode and a saturated calomel electrode (SCE) as a reference. After purging N_2_ gas for 30 min, a 1 m KOH electrolyte was employed as the electrolyte. The LSV data were recorded at a scan rate of 5 mV s^−1^ with a rotating speed of 1600 rpm (RRDE, ALS‐Co.). The measured potentials were normalized with respect to the reversible hydrogen electrode (RHE) based on the following equation: E_RHE_ = E_SCE_ + 1.0464 V (for 1 m KOH). Tafel slopes were determined by plotting the overpotential (η) as a function of log (j). The ECSA was evaluated from the electrochemical double‐layer capacitance. The TOF values were calculated at an overpotential of 400 mV according to a previously reported method.^[^ ^47^ ^]^ The measurement of EIS was conducted using an IVIUM analyzer in the 100 kHz−100 mHz frequency range.

### DFT Calculation

All DFT calculations were performed using the Vienna Ab initio Simulation Package (VASP).^[^ ^48^, ^49^ ^]^ We described the exchange‐correlation potential using the Perdew−Burke−Ernzerhof (PBE) functional within the generalized gradient approximation (GGA).^[^ ^50^ ^]^ The electron−ion interactions were treated with the projector augmented wave (PAW) method.^[^ ^51^ ^]^ A cutoff energy of 450 eV was set for the plane‐wave basis, and a 2 × 2 × 1 Monkhorst‐Pack k‐point mesh was used to sample the Brillouin zone. The calculations were considered converged when the energy change between self‐consistent iterations was below 10^−5^ eV and the forces on atoms were less than 0.05 eV Å^−1^. To account for van der Waals interactions, we applied the DFT‐D3 dispersion correction method.^[^ ^52^ ^]^

The Gibbs free energy changes (ΔG) for the reactions were calculated using the following equation:

(1) $$\Delta G=\Delta E+\Delta ZPE-T\Delta S+\Delta G_{U}+\Delta G_{pH}$$

where ΔE is the electronic energy difference directly obtained from the DFT calculations, ΔZPE is the zero‐point energy difference, T is the temperature (298.15 K), and ΔS is the entropy change. The term ΔG_U_ = −*eU* accounts for the effect of the applied electrode potential U, and Δ*G*_pH_ = *k*_B_ *T* × ln (10) × pH, where *k*_B_ is the Boltzmann constant.

## Conflict of Interest

The authors declare no conflict of interest.

## Supporting information



Supporting Information

## Data Availability

The data that support the findings of this study are available from the corresponding author upon reasonable request.
