# Supplementary material for: Design Rule for Highly Stable Efficient High‐Entropy Metal Oxide Electrocatalysts: Complementary Roles of 3d Transition Metal Ions
Source: Adv Sci (Weinh). 2025 Sep 16;12(45):e10594. doi: 10.1002/advs.202510594 (PMC12677678; doi:10.1002/advs.202510594)
Supplement: Supplementary file 1 — Supporting Information [file ADVS-12-e10594-s001.docx]

Supporting Information

Design Rule for Highly Stable Efficient High-Entropy Metal Oxide Electrocatalysts: Complementary Roles of 3d Transition Metal Ions

Nam Hee Kwon, Woo Jin Noh, Seong-Ju Hwang,* and Xiaoyan Jin*

N. H. Kwon, S.-J. Hwang

Department of Materials Science and Engineering, College of Engineering, Yonsei University, Seoul 03722, Republic of Korea

E-mail: hwangsju@yonsei.ac.kr

S.-J. Hwang

Department of Battery Engineering, Yonsei University, Seoul 03722, Republic of Korea

W. J. Noh, X. Jin

Department of Applied Chemistry, University of Seoul, Seoul 02504, Republic of Korea

E-mail: xjin@uos.ac.kr


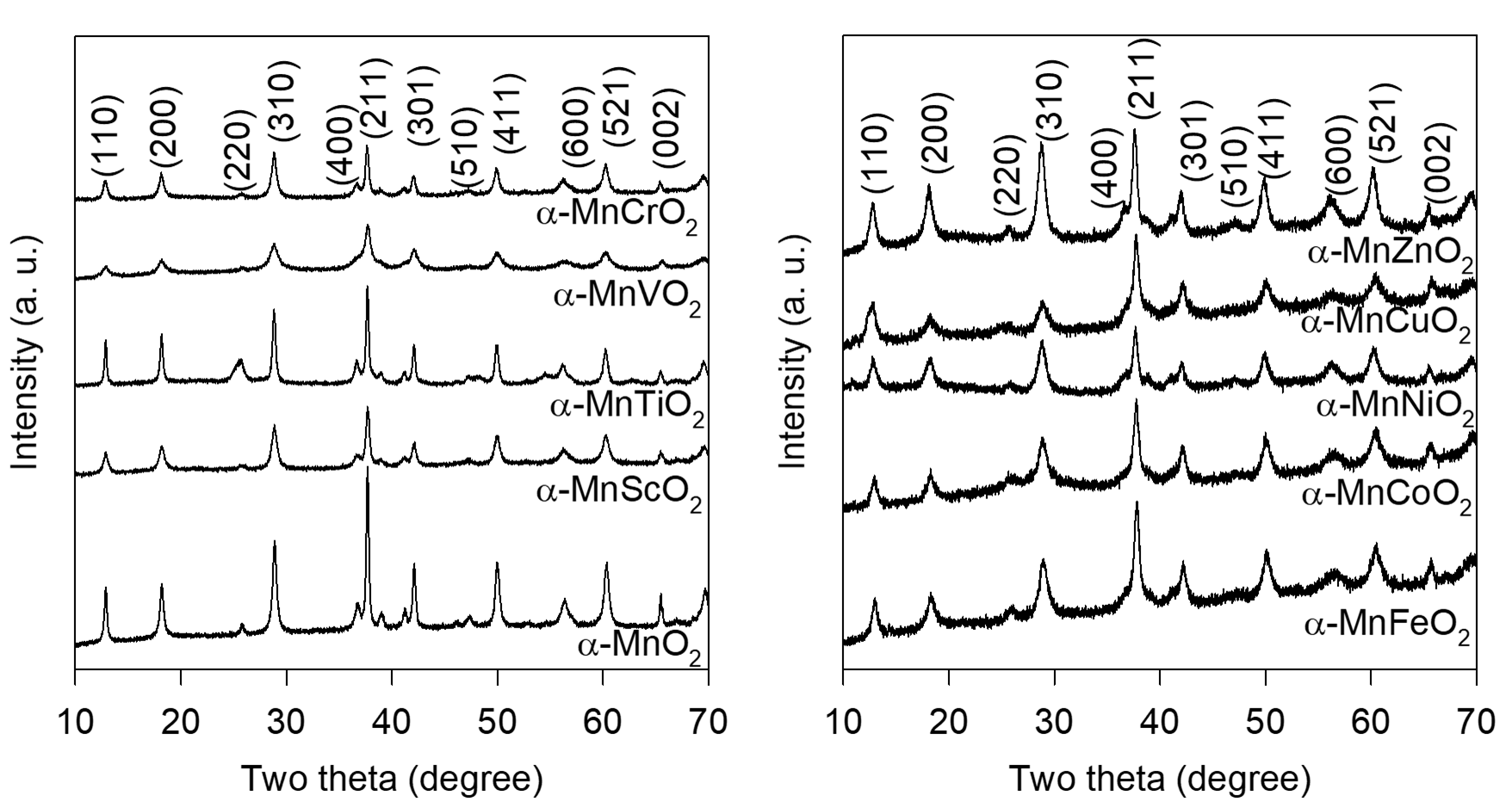


**Figure S1.** Powder X-ray diffraction (XRD) patterns of 3d transition metal ion substituted α-Mn_1−x_M_x_O_2_.


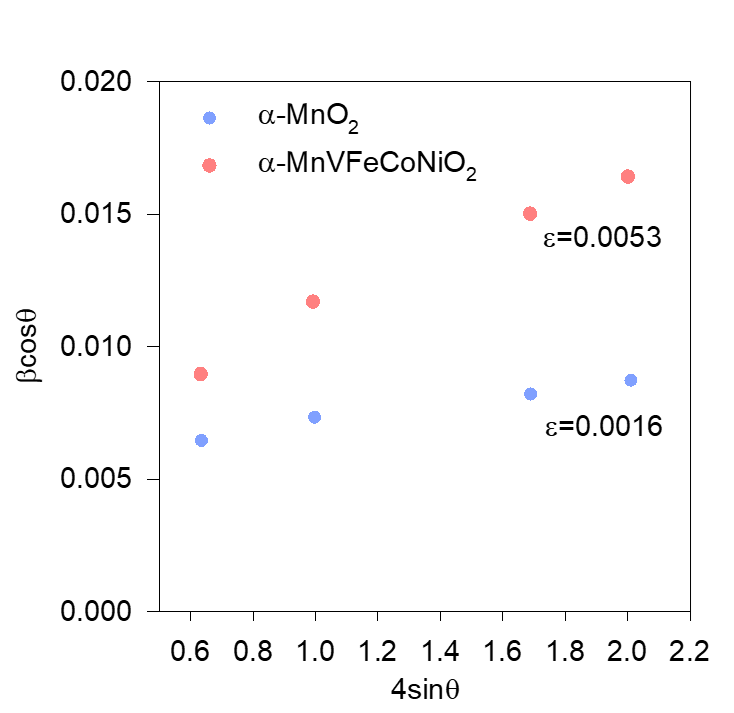


**Figure S2.** Lattice strain data of α-MnO_2_ and α-MnVFeCoNiO_2_.


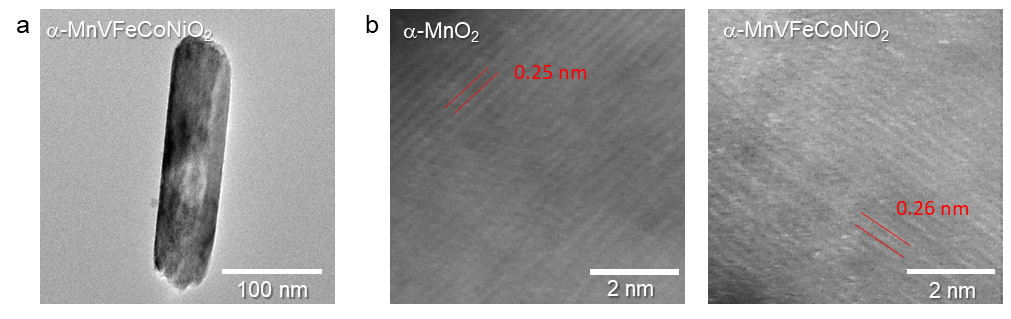


**Figure S3.** (a) Transmission electron miscroscopy (TEM) and high resolution transmission electron microscopy (HRTEM) images of α-MnO_2_ and α-MnVFeCoNiO_2_.


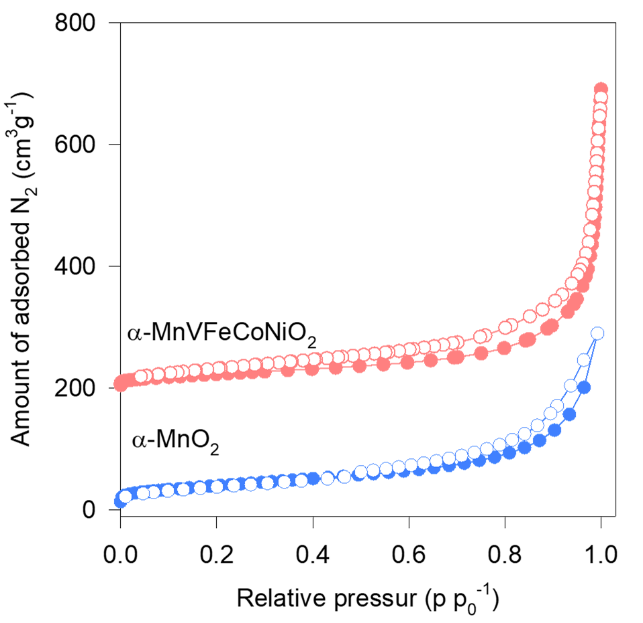


**Figure S4.** N_2_ adsorption−desorption isotherm curves of α-MnO_2_ and α-MnVFeCoNiO_2_.


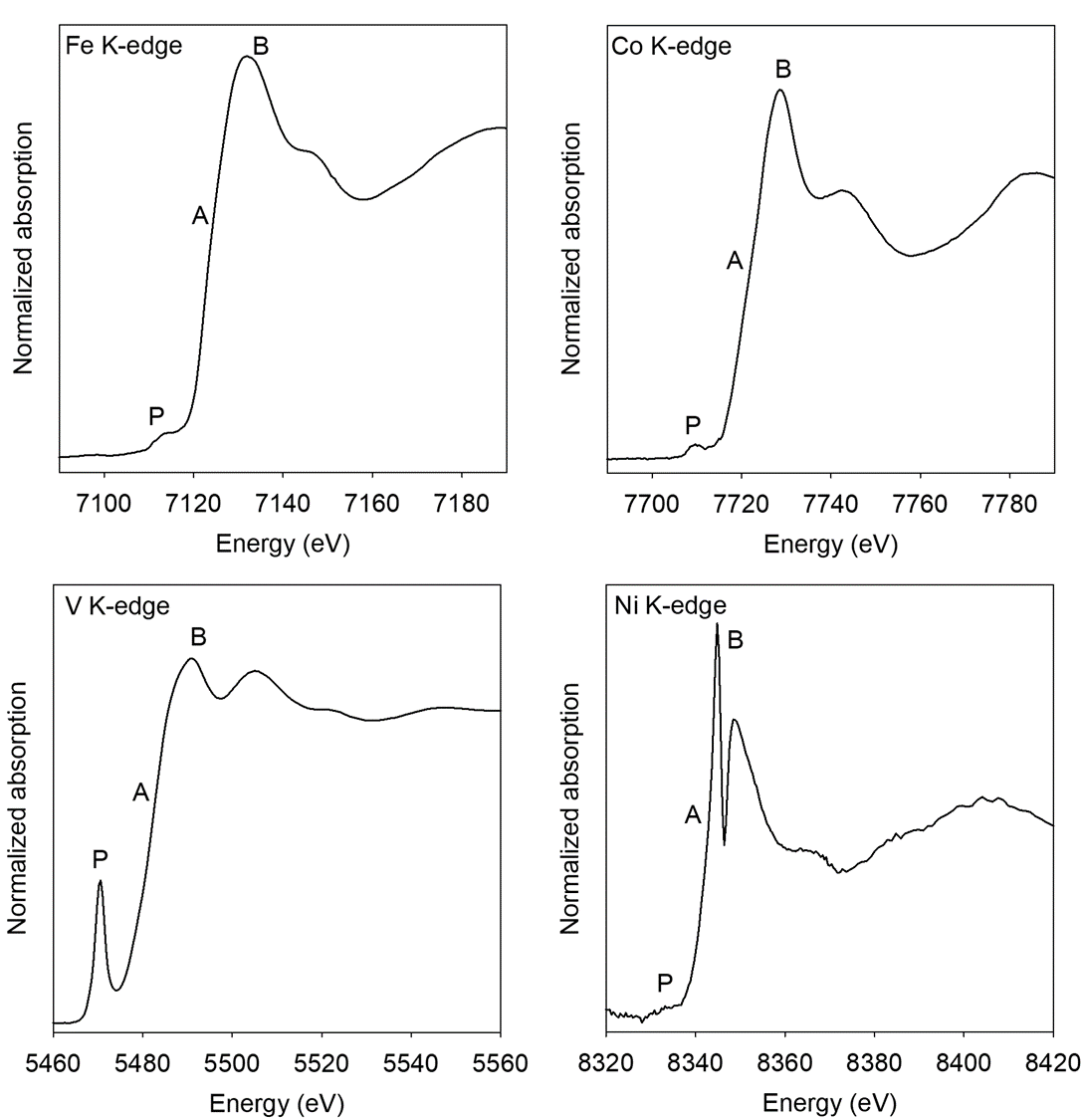


**Figure S5.** X-ray absorption near edge structure (XANES) data of binary α-MnFeO_2_, α-MnCoO_2_, α-MnVO_2_, and α-MnNiO_2_.


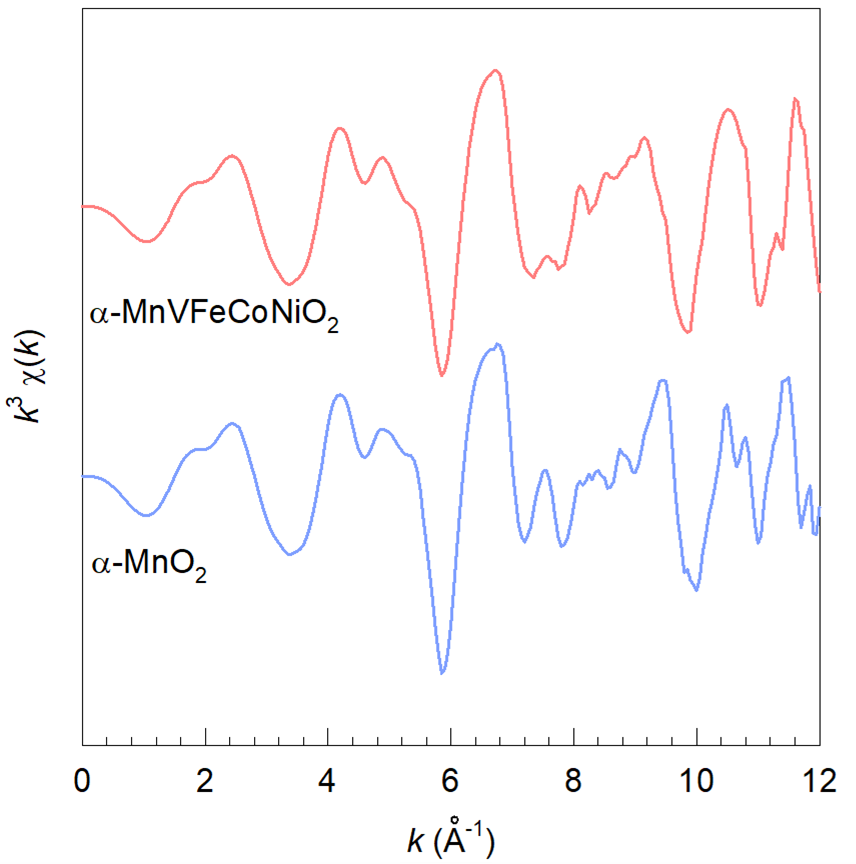


**Figure S6.** *k*^3^-weighted Mn K-edge extended X-ray absorption fine structure (EXAFS) oscillations of α-MnO_2_ and α-MnVFeCoNiO_2_.


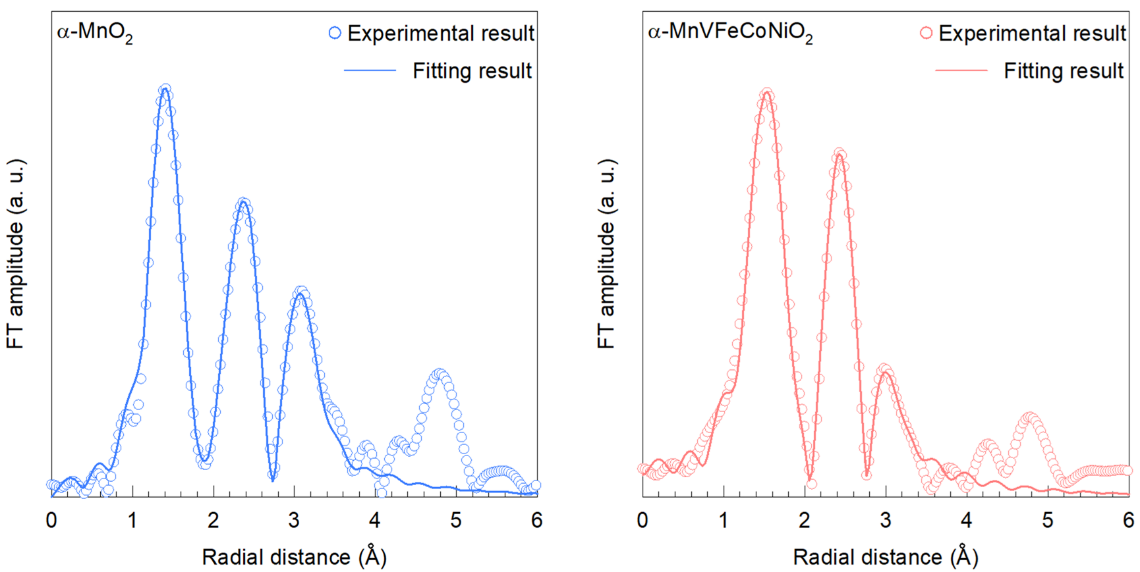


**Figure S7.** Mn K-edge EXAFS fitting analysis data of α-MnO_2_ and α-MnVFeCoNiO_2_.

**
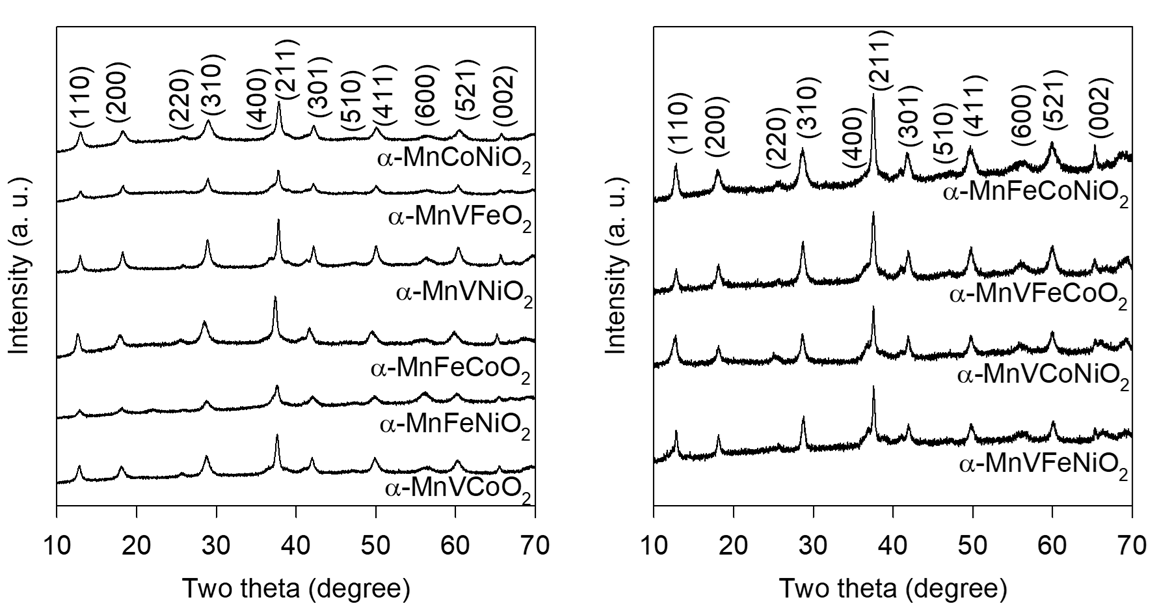
**

**Figure S8.** Powder X-ray diffraction (XRD) patterns of binary/ternary/quaternary metal-based α-MnO_2_.


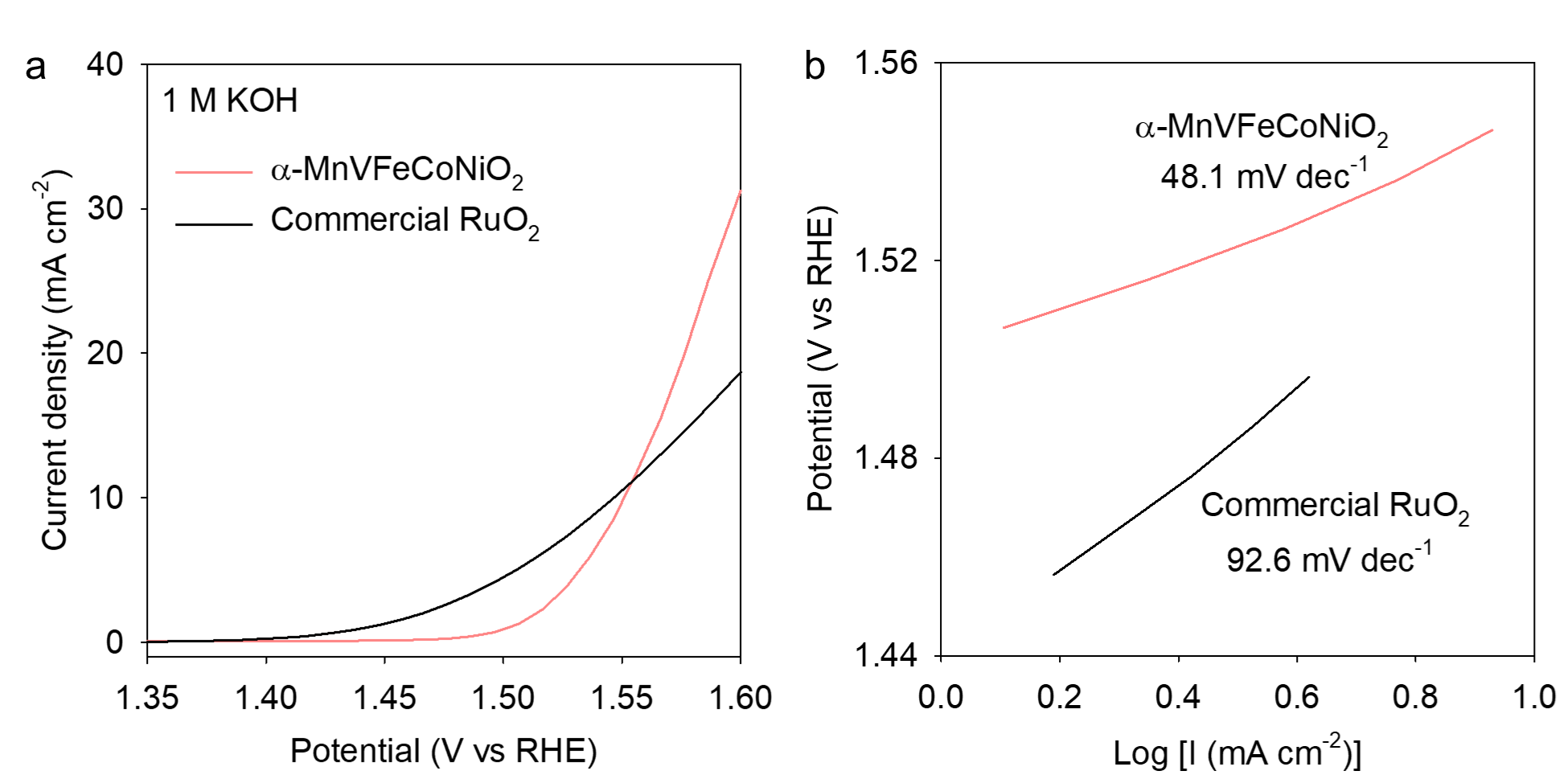


**Figure S9.** (a) OER polarization curves and (b) Tafel plots for α-MnVFeCoNiO_2_ and commercial RuO_2_.


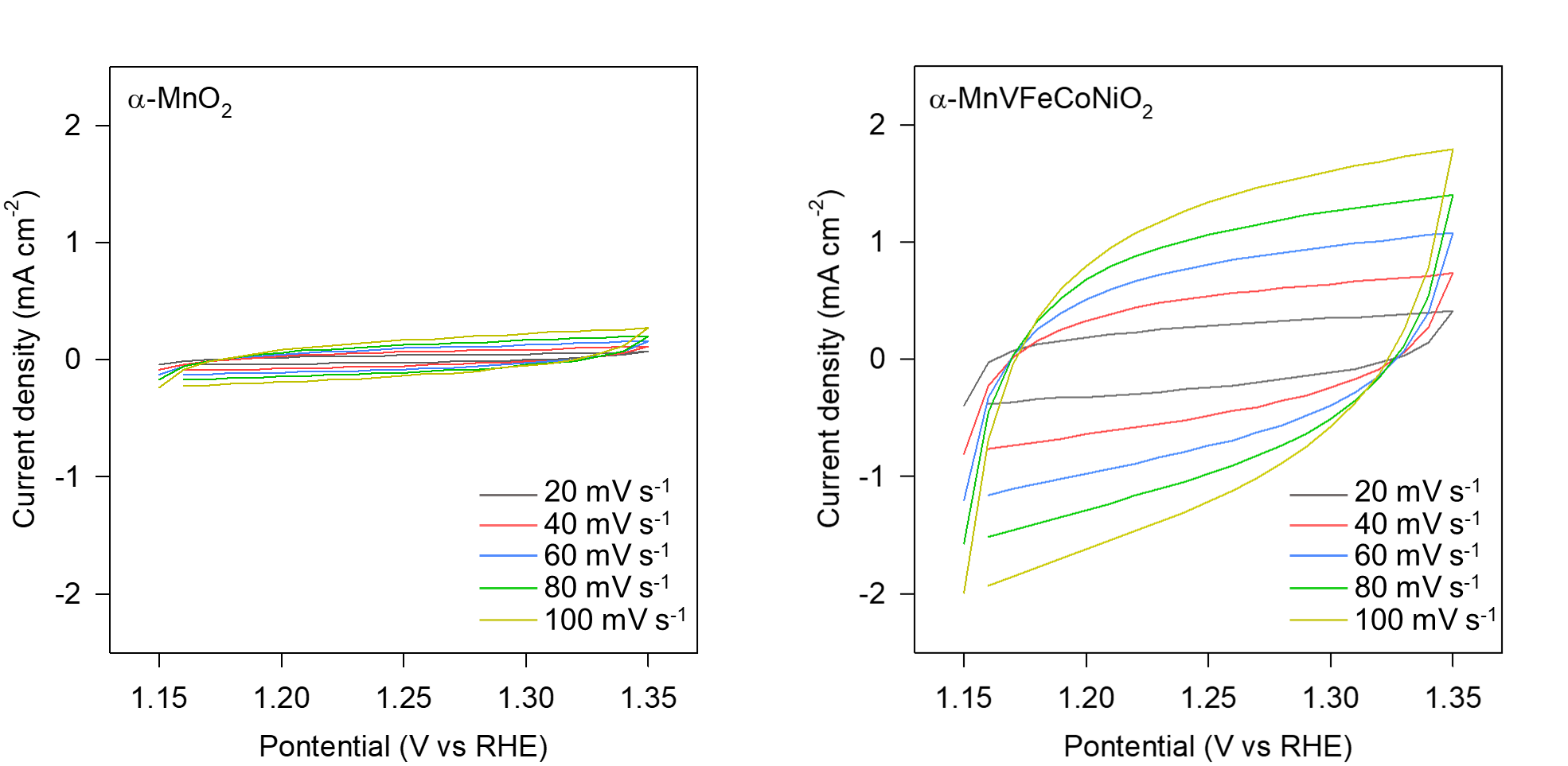


**Figure S10.** Cyclic voltammetry (CV) curves of α-MnO_2_ and α-MnVFeCoNiO_2_ measured at various scan rates.

**
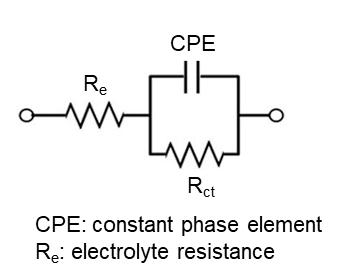
**

**Figure S11.** The equivalent circuit used to analyze the electrode−electrolyte interface.


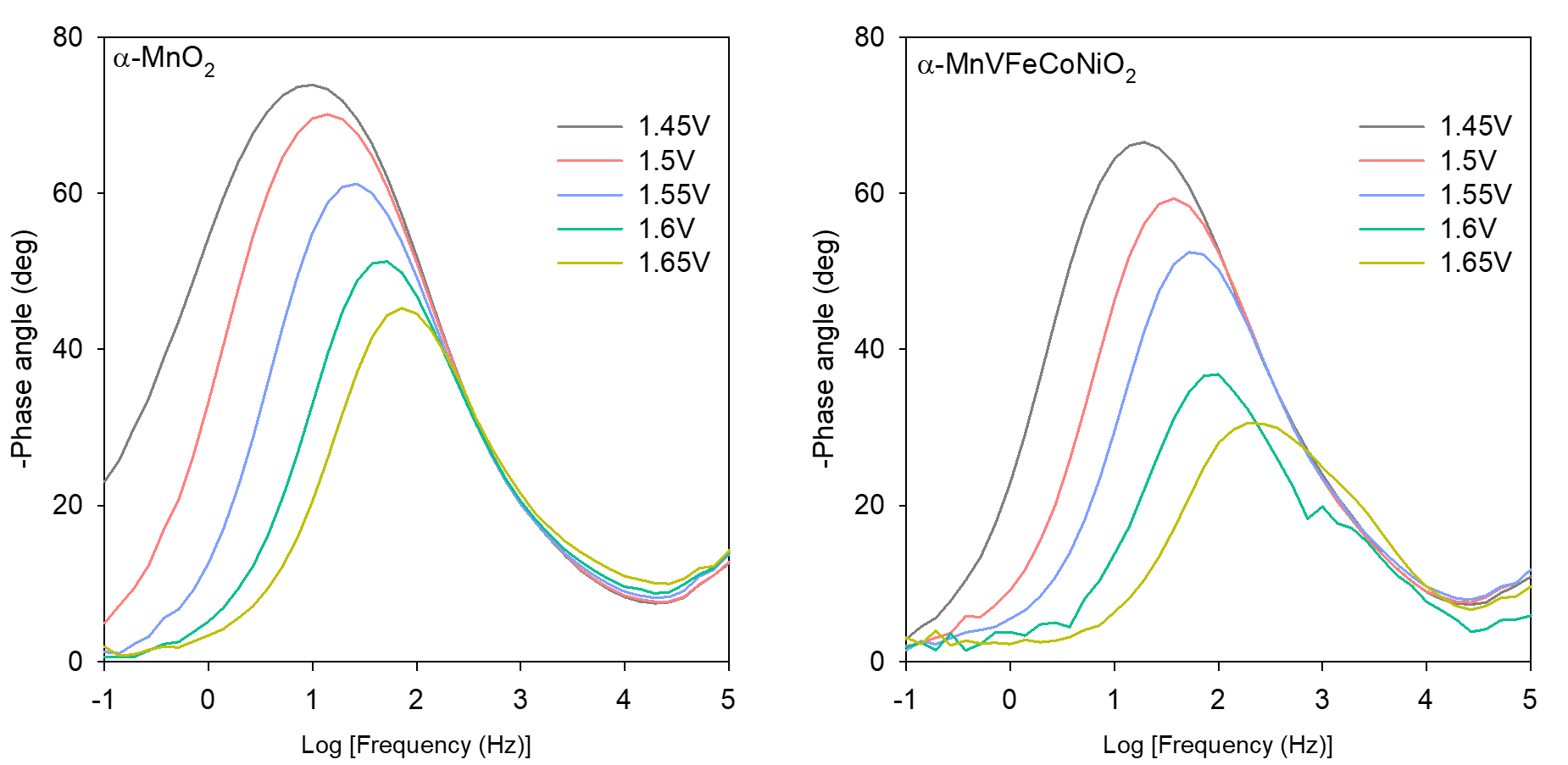


**Figure S12.** In situ Bode plots of α-MnO_2_ and α-MnVFeCoNiO_2_.


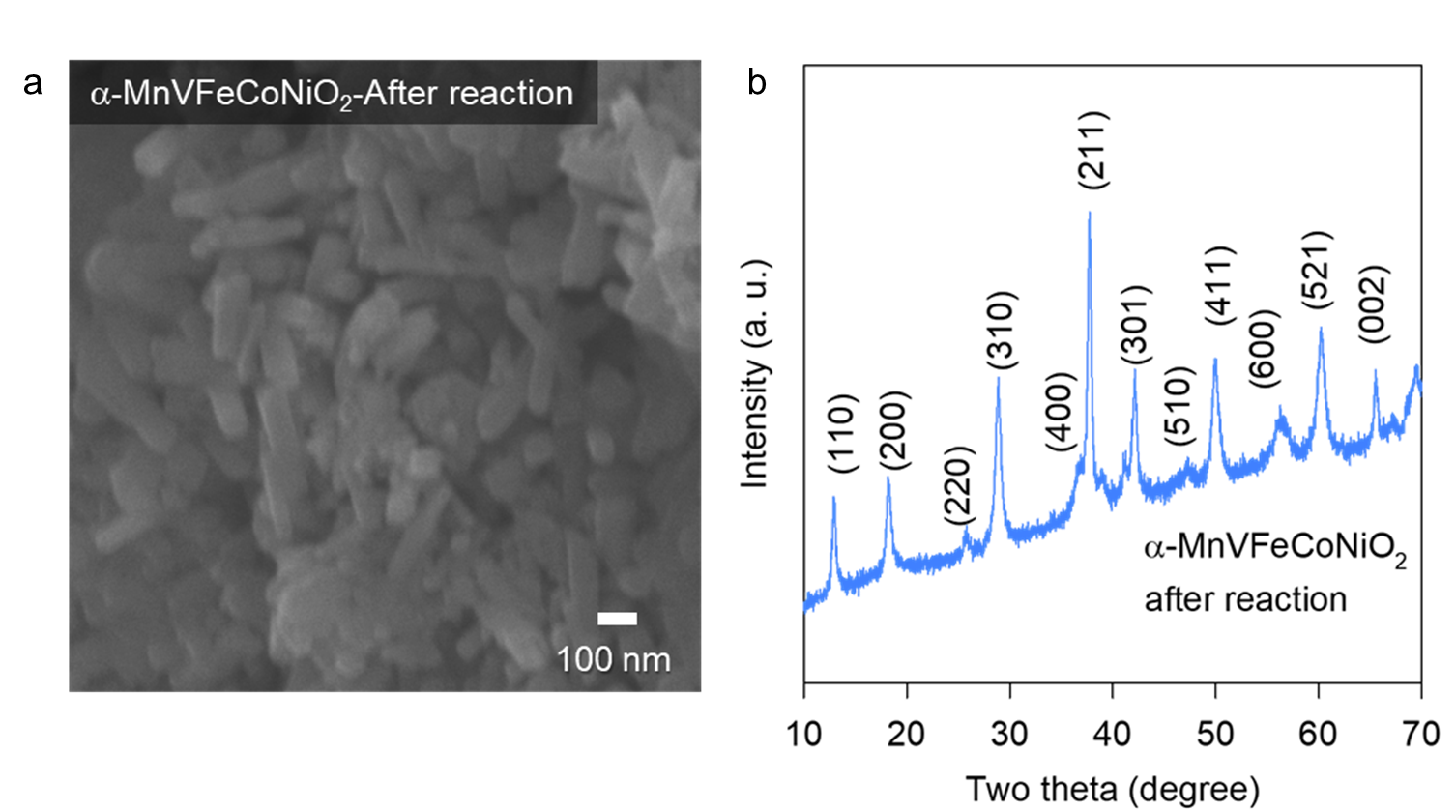


**Figure S13.** (a) Field emission-scanning electron microscopy (FE-SEM) image and (b) powder X-ray diffraction (XRD) pattern of α-MnVFeCoNiO_2_ after long-term OER process.


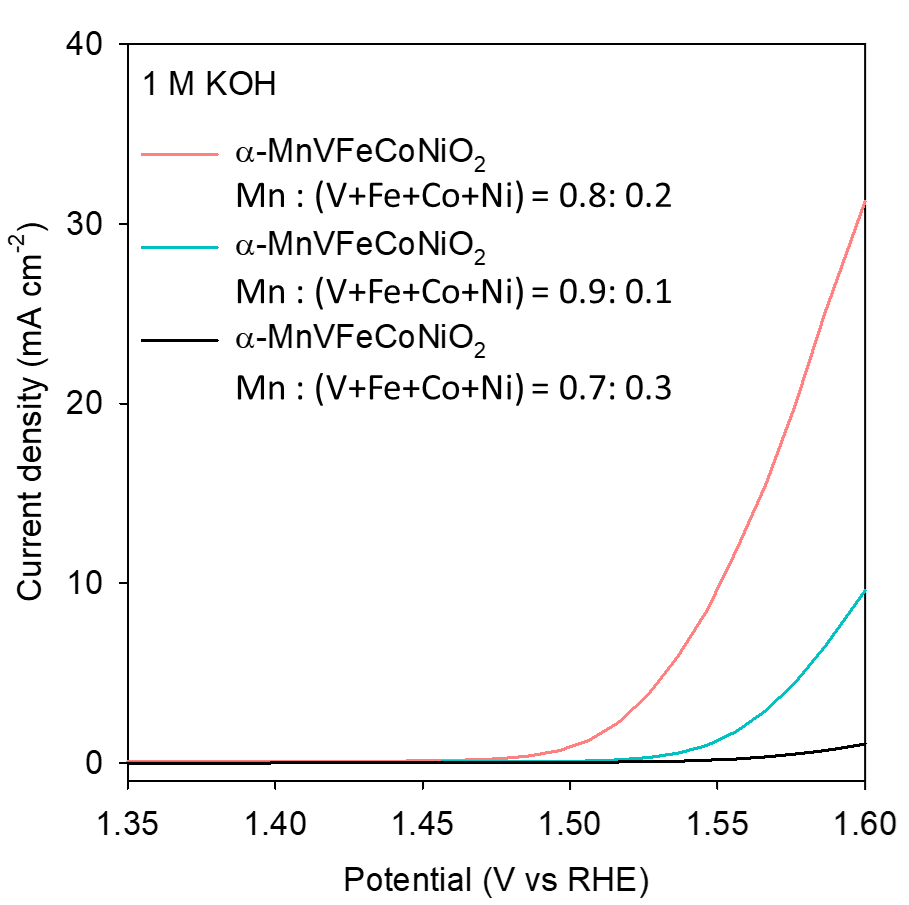


**Figure S14.** Linear sweep voltammetry (LSV) curves of α-MnVFeCoNiO_2_ materials with different Mn, V, Fe, Co, and Ni ratios.

**Table S1.** Lattice parameters of α-MnO_2_ and α-MnVFeCoNiO_2_.

| **Material** | **a (Å)** | **c (Å)** |
| --- | --- | --- |
| α-MnO_2_ | 9.783 | 2.852 |
| α-MnVFeCoNiO_2_ | 9.801 | 2.855 |

**Table S2.** Relative concentrations of Mn species in the materials determined from Mn 2p_3/2_ X-ray photoelectron spectroscopy (XPS) data of α-MnO_2_ and α-MnVFeCoNiO_2_.

| **Material** | **Mn^3+^** | **Mn^4+^** |
| --- | --- | --- |
| α-MnO_2_ | 47.8% | 52.2% |
| α-MnVFeCoNiO_2_ | 63.4% | 36.6% |

**Table S3.** Results of non-linear least-squares Mn K-edge EXAFS fitting analysis of α-MnO_2_ and α-MnVFeCoNiO_2_.

| Material | Bonding pair | CN | R (Å) | ΔE (eV) | σ^2^ (Å^2^) |
| --- | --- | --- | --- | --- | --- |
| α-MnO_2_ | Mn−O  Mn−Mn  Mn−Mn | 6  4  4 | 1.904  2.873  3.451 | 1.26  −4.85  −1.35 | 0.0024  0.0084  0.0042 |
| α-MnVFeCoNiO_2_ | Mn−O  Mn−Mn  Mn−Mn | 6  4  4 | 1.923  2.890  3.421 | 3.39  −2.54  −2.99 | 0.0071  0.0091  0.0056 |

**Table S4.** Comparison of the electrocatalytic activity with transition metal doped MnO_2_-based catalysts toward OER.

| Material | Electrolyte | Overpotential  (10 mA cm^−2^) | Tafel slope (mV dec^−1^) | ref |
| --- | --- | --- | --- | --- |
| Ni doped MnO_2_ | 0.1 M KOH | 445 | 86 | [1] |
| Fe-doped MnO_2_/NF | 1 M KOH | 330 | 51 | [2] |
| Ni doped α-MnO_2_ | 0.1 M KOH | 366 | 160 | [3] |
| Fe-doped α-MnO_2_ | 1 M KOH | 400 | 59 | [4] |
| V-doped α-MnO_2_ | 1 M KOH | 536 | 150 | [5] |
| Co-doped α-MnO_2_ | 1 M KOH | 506 | 120 | [5] |
| Ti doped MnO_2_ | 1 M KOH | 410 | 145 | [6] |
| Fe,V,Co,Ni doped MnO_2_ nanosheets | 1 M KOH | 390 | 104 | [7] |
| α-MnVFeCoNiO_2_ | 1 M KOH | 321 | 48.1 | This work |

**Table S5.** Metal and material prices of the present materials.

| **Material** | **Approximate prices as of August 2025 (USD/kg)** |
| --- | --- |
| Mn | 2 |
| V | 185 |
| Co | 33 |
| Ni | 15 |
| Fe | 0.1 |
| Ru | 25700 |
| Ir | 151108 |
| Ru (20%) substituted α-MnO_2_ | 5141 |
| Ir (20%) substituted α-MnO_2_ | 120888 |
| α-MnVFeCoNiO_2_ | 13.2 |

**References**

‌[1] K. Bera, A. Karmakar, K. Karthick, S. S. Sankar, S. Kumaravel, R. Madhu, S. Kundu, *Inorg. Chem.* **2021**, *60*, 19429.

[2] J. Lu, H. Wang, Y. Sun, X. Wang, X. Song, R. Wang, *Chem. Eng. J.* **2021**, *417*, 127894.

[3] J. Xie, Y. Chen, Z. He, S. Liu, Y.L. Liu, B. Li, T. Xu, X. Ning, S. Chen, T. Zeng, H. He, *ACS Appl. Nano Mater.* **2024**, *7*, 18027.

[4] J. M. Lee, S. B. Patil. B. Kang, S. Lee, M. G. Kim, S.-J. Hwang, *J. Mater. Chem. A* **2018**, *6*, 12565.

[5] J. M. Lee, S.-J. Hwang, *J. Solid State Chem.* **2019**, *269*, 354.

[6] Y. Liu, S. Ma, S. Zhang, F. Liu, Y. Wang, X. Sun, Y. Li, Y. Xue, C. Tang, J. Zhang, *Fuel* **2024**, *374*, 132424.

[7] Z. Ye, T. Li, G. Ma, Y. Dong, X. Zhou, *Adv. Funct. Mater.* **2017**, *27*, 1704083.
